# Action Potential Waveform Variability Limits Multi-Unit Separation in Freely Behaving Rats

**DOI:** 10.1371/journal.pone.0038482

**Published:** 2012-06-12

**Authors:** Peter Stratton, Allen Cheung, Janet Wiles, Eugene Kiyatkin, Pankaj Sah, François Windels

**Affiliations:** 1 Queensland Brain Institute, The University of Queensland, Brisbane, Queensland, Australia; 2 School of Information Technology and Electrical Engineering, The University of Queensland, Brisbane, Queensland, Australia; 3 Behavioral Neuroscience Branch, National Institute on Drug Abuse - Intramural Research Program, National Institutes of Health, Department of Health and Human Services (DHHS), Baltimore, Maryland, United States of America; University of New South Wales, Australia

## Abstract

Extracellular multi-unit recording is a widely used technique to study spontaneous and evoked neuronal activity in awake behaving animals. These recordings are done using either single-wire or mulitwire electrodes such as tetrodes. In this study we have tested the ability of single-wire electrodes to discriminate activity from multiple neurons under conditions of varying noise and neuronal cell density. Using extracellular single-unit recording, coupled with iontophoresis to drive cell activity across a wide dynamic range, we studied spike waveform variability, and explored systematic differences in single-unit spike waveform within and between brain regions as well as the influence of signal-to-noise ratio (SNR) on the similarity of spike waveforms. We also modelled spike misclassification for a range of cell densities based on neuronal recordings obtained at different SNRs. Modelling predictions were confirmed by classifying spike waveforms from multiple cells with various SNRs using a leading commercial spike-sorting system. Our results show that for single-wire recordings, multiple units can only be reliably distinguished under conditions of high recording SNR (≥4) and low neuronal density (≈20,000/ mm^3^). Physiological and behavioural changes, as well as technical limitations typical of awake animal preparations, reduce the accuracy of single-channel spike classification, resulting in serious classification errors. For SNR <4, the probability of misclassifying spikes approaches 100% in many cases. Our results suggest that in studies where the SNR is low or neuronal density is high, separation of distinct units needs to be evaluated with great caution.

## Introduction

Extracellular multi-unit recording of neural activity is widely used to study spontaneous or evoked neuronal activity in anaesthetised as well as awake behaving animals. The ability to simultaneously differentiate distinct neurons in multiple regions has provided detailed insights into the neural representation of sensory information in the mammalian brain as well as the functional roles of local and projection circuits. Clearly, in such studies the accurate isolation of multiple distinct single units for each electrode is crucial for correct interpretation of the recorded data. Although action potentials propagate into the dendritic tree and axon, modelling studies indicate that the origin of the action potential waveform recorded by these electrodes is likely to be the soma [1], [2] as it generates the largest signal compared to other sources along the neuronal processes. An electrode in extracellular space is therefore expected to sample activity originating from a number of surrounding active neurons. Electrical signals in the brain decay with increasing distance between the source and the electrode, approximately following an inverse square law [3]. The maximum distance separating a recordable unit from an electrode can therefore be estimated, and the number of cells within this range calculated [4]. Analytical tools have been developed to process such recorded activity and assign isolated spikes to specific units [4], [5], [6]. However, accurate classification remains difficult when large numbers of cells are present around the recording electrode [5], [6], a situation in which the misclassification of recorded units becomes more likely.

This problem of misclassification in layered brain regions (where neuronal density is relatively high) was markedly improved by the development of multi-wire recording electrodes [7]. In the case of tetrodes, for example, spike sorting is based on a four-wire recording of the same unit, where the relative distance between each wire and the action potential source produces a different signal on each channel. This unique set of waveforms, related to the cell’s position, allows for the accurate isolation of multiple single units, as well as the possibility of tracking of multiple units over many days. However, this method also has two major limitations: 1) each recording site requires four recording channels, with the consequent increase in the complexity of the experiment and the volume of data collected per electrode; and 2) the relatively large total diameter and coarse tips of the multiple wires comprising the tetrode can cause increased damage to the neural tissue through which it passes. For these reasons, single electrodes are still predominantly used in those brain regions where neurons are not very densely packed (for example, most regions outside the hippocampus and some cortical layers).

For lower density brain regions, unit sorting is often done using a combination of template matching and autocorrelation of spike times recorded using twisted electrode bundles [8]–[10]. This technique is based on the assumptions that a) spikes from different neurons will have different apparent waveforms because of variations in the spatial configuration between the neuron and the electrode tip and b) the average spike amplitude between cells will differ because it is unlikely that two neurons will be at the same distance from the electrode tip, meaning that distinct units will be clearly separable. Both of these assumptions require that spike shape and amplitude in individual cells are stable. This, however, is clearly not the case. For example, Fee and colleagues [11] showed that spike amplitude changes over the course of a single continuous recording, with spike size decreasing at higher firing rates. Action potential shape also changes with activity, thereby affecting the measured duration of the spikes [12]. Moreover, the impact of these two factors on the quality of the spike sorting is exacerbated by fluctuations in the signal-to-noise ratio (SNR) or the presence of a nearby cell with similar spike shape. The latter could have a particularly strong impact considering that signals originating from a neuron spread isotropically in the neuropil [13], and the fact that the recorded waveform originates mainly from the soma [1], [2]. As a consequence, there would be a critical volume around the electrode within which two different cells could be falsely identified as a single unit. To date, however, no study has investigated the theoretical and practical limits of spike waveform separability given different recording noise levels, different numbers of recorded neurons, or the presence of similar spike waveforms from different neurons.

In this paper we analysed single-unit recordings in unrestrained animals (anaesthetised and non-anaesthetised) using small diameter multi-barrel glass pipettes (<5 µm) placed in a one-axis drive [14]. This method allows the position of the electrode to be accurately controlled while monitoring the neural activity, thereby allowing improvement of the recording’s SNR. The high impedance of these electrodes, together with the on-line adjustment of position, makes this method ideal for isolating the signal of single units in unrestrained non-anaesthetised animals [15]–[17]. Multi-barrel electrodes were used for iontophoretic application of gamma amino-butyric acid (GABA) and glutamate [18] to induce neural activity changes over a wide dynamic range. Such manipulations, which mimic the changes in activity that are observed *in vivo,* were used to test spike waveform variability over a wide range of firing frequencies. We assessed systematic differences in single-unit spike waveforms within and between brain regions (substantia nigra, mesencephalon and tectum) with differing cell densities, soma sizes and neurochemical content [19]–[23]. We also investigated the influence of SNR on spike waveform characteristics and modelled spike misclassification for different cell densities based on neuronal recordings at different SNRs. We aimed to specifically test the ability of a single electrode to discriminate activity from multiple neurons. Our analyses show that using single recording electrodes, multiple units can only be reliably distinguished under favourable conditions of high recording SNR and low neuronal density.

## Methods

### Animals and Surgery

Data were obtained from 26 male Long-Evans rats (400±50 g) supplied by Charles River Laboratories (Greensboro, NC, USA). All animals were housed individually under standard laboratory conditions (12-hr light cycle beginning at 07:00) with free access to food and water. Protocols were performed in compliance with the National Institutes of Health Guide for the Care and Use of Laboratory Animals and were approved by the National Institute of Drug Abuse Animal Care and Use Committee. The surgical procedures used have been described previously [18]. Briefly, under general anaesthesia (Equithesin 0.33 ml/100 g i.p.; dose of sodium pentobarbital 32.5 mg/kg and chlorale hydrate 145 mg/kg), rats were implanted with a plastic, cylindrical hub, designed to mate with a microelectrode holder during recording [14]. This hub was centred over a hole drilled above the substantia nigra pars compacta and pars reticulata, the deep mesencephalic nucleus, or the anterior pretectal nucleus. After a 3–4 day recovery period and habituation to the experimental chamber, recording sessions were held once daily over 1–3 days. A separate group of rats (n = 5), prepared as described above, underwent a recording session under chloral hydrate anaesthesia (400 mg/kg, i.p., followed by 120 mg/kg/hr). In these experiments body temperature was maintained automatically at 37.2±0.2°C with an electric heating pad and feedback rectal thermal probe.

### Electrophysiology and Iontophoresis

Four-barrel, microfilament-filled, glass pipettes (Omega Dot 50744, Stoelting, Wood Dale, IL, USA), pulled and broken to a diameter of 5±1 µm, were used for single-unit recording and iontophoresis. The recording barrel contained 2% pontamine sky blue (BDH Chemicals Ltd, Poole, UK) in 3 M NaCl and the balance barrel contained 0.25 M NaCl. The remaining barrels were filled with solutions of l-glutamate (0.25 M in distilled water, pH 5.5; Sigma, St Louis, USA) or GABA (0.25 M in 0.125 M NaCl water, pH 4.5, Sigma). The resistance of the recording channel was 3–5 MΩ (measured at 100 Hz) and that of the drug-containing barrels ranged from 10–35 MΩ. Retaining (–8 to –10 nA) and ejecting (+20 to 40 nA) currents were applied with a constant current generator (Ion 100T, Dagan, Minneapolis, MN, USA). Each multibarrel pipette was filled with fresh solution less than one hour before use and fixed in a microdrive assembly that later was inserted into the skull-mounted hub. The electrode was then advanced 3.0 mm below the brain surface to the starting point of unit recording.

**Figure 1:**
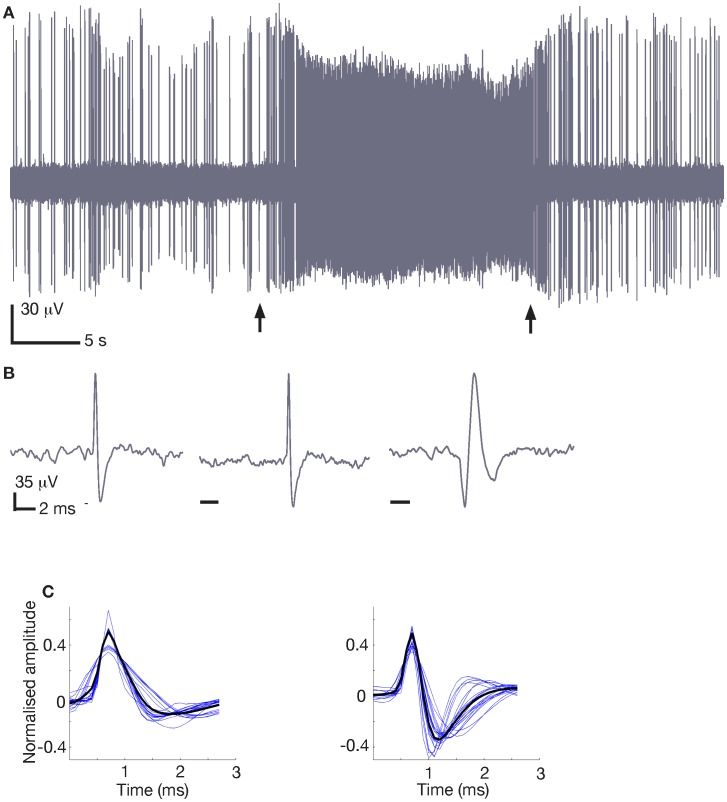
Example single-unit recording and spike waveforms. A : Single-unit activity recorded in the anterior pretectal nucleus of an unanaesthetised, unrestrained rat in during iontophoretic application of glutamate (between arrows). **B**: Individual spikes recorded from three different cells in the anterior pretectal nucleus (left, same recording as A), the substantia nigra pars reticulata (middle) and a triphasic spike from the substantia nigra pars compacta (right). **C**: Spikes from single-wire, single-unit recordings are similar to spikes from multi-unit recordings. Left: The mean spikes from 20 randomly selected hippocampal CA1 neurons (blue) and the mean of all those means (black). Right: The mean spike shape for each of the 23 biphasic cells (blue) recorded and analysed using the single-unit protocol, and the mean of all the mean shapes (black). CA1 data are courtesy of the Buzsaki group from the Collaborative Research in Computational Neuroscience data-sharing website (crcns.org).

Neuronal discharge signals were sent to a head-mounted preamplifier (OPA 404KP, Burr Brown, Tucson, AZ, USA) and then further amplified and filtered (band pass: 300–3,000 Hz) with a Neurolog System (Digitimer, Welwyn Garden City, UK). The filtered signal was recorded using a Micro 1401 MK2 interface (Cambridge Electronic Design, Cambridge, UK). Spike activity was monitored with a digital oscilloscope and audio amplifier, and analysed using a Spike2 interface (Cambridge Electronic Design). Recordings to be analysed were selected based on several parameters: (i) there was a significant period of recording stability (stable SNR, see below for details), (ii) the cell exhibited a variety of firing rates during that period, and (iii) for some cells, firing rate was manipulated by iontophoretic applications of glutamate or GABA released by constant or increasing ejection currents for 20 s at 60 to 90 s intervals. Spike detection thresholds were set manually for each recording to ensure that an optimal number of spikes were extracted, this being particularly important for recordings with low SNR. For biphasic spikes, 2.75 ms of recording was extracted for each spike (0.75 ms before and 2.0 ms after each peak), while for triphasic spikes, 6.0 ms was extracted (2.0 ms before and 4.0 ms after). During the experiment the rat’s activity was recorded using a wide-angle camera (Creative Technology, Milpitas, CA, USA). All iontophoretic applications used for statistical analysis were performed when the animal was at rest with no sign of overt movements. An example of a typical single-unit recording with glutamate iontophoresis is shown in Figure 1A. Baseline activity and changes in spike amplitude can be observed before and after the iontophoresis. Glutamate was applied during the epoch marked by the two arrows, inducing a large increase in cell firing that correlated with a visible decrease in spike amplitude. When the same iontophoretic currents were used with solution containing no active compound, neither of these changes was observed, showing that iontophoretic currents were not directly responsible for the changes in cell activity or spike amplitude, consistent with other experimental findings [24]. Typical spike waveforms are illustrated in Figure 1B.

### Histology

After the last recording session, the rats were anaesthetised and pontamine sky blue was deposited by current injection (-20 mA for 20 min) at the last recording site. Animals were then perfused transcardially with saline solution followed by 4% paraformaldehyde. The brain was removed and placed overnight at 4°C in a 20% sucrose solution before being frozen in dry ice. Coronal 30 µm tissue sections were prepared at –20°C using a microtome cryostat. The Paxinos and Watson atlas [25] served as the basis for histological analyses.

### Spikes Waveform Analysis

The SNR of each recording was calculated as RMS_signal_/RMS_noise_ where the signal comprised all extracted spikes and the noise comprised the remainder of the recording. RMS is the root mean square of the data (*i.e.* √[(Σ*^n^x_i_* ^2^)/*n*], where *x_i_*, *i* = 1.n, is the complete set of data values) which, for data with zero mean (such as the band-pass filtered recordings), is equal to the standard deviation. For biphasic spikes, a total of 2.75 ms of recording was extracted for each spike, whereas for triphasic spikes, 6.0 ms was extracted, and the signal RMS was calculated on the full spike waveforms extracted for each recording (*i.e.* the RMS calculation was performed on longer sample periods for triphasic spikes). Noise RMS was calculated across the entire recording, less the extracted spikes, so may have included low-amplitude multi-unit activity as a component of the noise (*i.e.* spike waveforms, from nearby cells, that were too small to reach spike detection threshold, and which were occasionally visible in some recordings). Note that SNR is sometimes alternately measured as spike peak amplitude divided by RMS_noise_; such treatment of SNR yields values typically 2 to 2.5 times higher than defined here, depending on spike shape (see Figure S3 and Material S1 and S2 for more information). Spikes were detected with threshold crossing, after which detected spikes were 10× oversampled with spline interpolation and realigned on the spline spike peak. All subsequent operations on the spikes were conducted on the oversampled, realigned versions. The amplitude of each spike was calculated as the absolute total amplitude spanned by the spike waveform, which was usually the distance between the spike peak and subsequent trough.

The continuous firing rate, *r*, was calculated at time *t* of each spike with terms for exponential rise and decay based on the interspike intervals of preceding spikes:
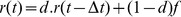
(1)where Δ*t* is the time since the preceding spike, *f* is the instantaneous firing rate (*f* = 1/Δ*t*) and *d* is the exponential decay term (*d*  =  *e* ^–Δt/τ^) with the exponential time constant τ = 100 ms [11] (approximating the cell membrane time constant). The decay term gives more weight to the instantaneous firing rate *f* after a long time delay since the previous spike (during which the membrane has had time to recover), while giving more weight to the slower-changing continuous firing rate during spike bursts (meaning that continuous firing rate *r* rises only slowly during bursts).

All the spikes extracted from a recording period were averaged (after aligning on the spike peak) to give a mean spike shape for that recording. The mean spike was then processed to identify important properties of the spike waveform. These points were the first and second zero crossings where net current flow was zero (points z_1_ and z_2_), the start point of the spike (point s), the spike half width (h), and the spike rise (r  =  z_1_–s) and fall times (f  =  z_2_–z_1_). The first zero crossing after the peak of the recorded extracellular spike was considered to be the spike peak of the intracellular spike [26]. The second zero crossing of the extracellular spike was considered to be close to the bottom of the intracellular spike trough, although this point may not be exact [26]. The extracellular spike half width (h) was calculated by first finding the maximum absolute value of the waveform prior to the first zero crossing. The first two points in the spike with amplitude of half this value were then identified, and the time difference between these points was taken as the half width. To determine the start point of the spike, an initial estimate was made by subtracting the half width from the extracellular spike (EC) peak position. A pre-spike recording baseline level was then calculated for each spike by finding the mean amplitude of the extracted spike prior to the estimated start point. The spike start point was then deemed to be the first point prior to the peak that crossed 5% above the baseline level (*i.e.* 5% of the height of the peak). This technique accommodated for spikes that did not start from a baseline level of exactly zero.

All spikes in the recording were sorted based on their calculated continuous firing rate and divided into 5 firing rate bins (if required, up to 4 spikes were omitted to ensure that bins were equally sized, with the average dataset being 2861 total spikes). Three methods were used to test for a correlation between spike amplitude and firing rate: (i) Bonferroni-corrected t-tests between the bins (and a separate final test between the first and last bins), (ii) one-way analyses of variance (ANOVA) across all bins, and (iii) determination of the line of best fit for all points on the spike amplitude *vs.* firing rate graph, with subsequent calculation of whether the 99% confidence interval (CI) for the slope of the line included zero (meaning that no significant amplitude *vs.* firing rate correlation existed). The spike waveform properties (s, z_1_, z_2_, h, r and f as described above) were then calculated as above for each individual spike, and one-way ANOVA tests were used to test for changes in rise times, fall times and half widths across bins.

### Heat Map Construction

All spike vectors were normalised to zero-mean and unit length. Each spike in a recording was then compared to the mean spike for that recording using the dot product (bearing in mind that spike peaks were aligned as part of the spike waveform analysis). The mean and standard deviation of the distribution of dot product values for all spikes in a recording provided a measure of the variability of the spike waveform from spike to spike, independent of any amplitude changes (since the spike vectors were normalised). Every spike for each recording was then also compared to the mean spike for every other recording using the same normalised dot product; this comparison provided a measure of the similarity between spikes from each neuron and the mean spikes from all other neurons. Large variability in spike waveforms due to noise meant that many spikes from some neurons were more similar to the spikes from other neurons, and hence would be confused with the other neurons for spike-sorting purposes. The results of these comparisons were plotted on an n*n grid with rows and columns labelled with the set of all recorded neurons. At each grid cell which marked the intersection of two given neurons, the cell was coloured based on how many of one cell’s spikes would be erroneously attributed to the other cell; such a representation is called a heat map and provides a concise graphical summary of all the comparisons.

### Hybrid Recording Construction

To test our spike sortability predictions on recordings with known neuron identities, hybrid recordings were created by concatenating spike waveforms from several neurons. Three such hybrid recordings were created with high, moderate and low SNR. Each recording contained 500 spikes from one neuron followed by 500 spikes from another. The hybrid recording was exported into *wave* format (.wav file) and then imported into Plexon Offline Sorter (OFS, Plexon, Dallas, USA). Two different spike sorting techniques were used: K-means scan sorting and valley-seeking sorting. Both techniques attempt to simultaneously determine the number of different clusters (neurons) in the data and to assign the spikes to those clusters. In the case of K-means sorting in OFS, different numbers of clusters can be returned by different cluster quality metrics, in which case a voting mechanism was employed to select the number of clusters for which most cluster metrics agreed. After sorting, the number of correctly classified spikes, together with the number of false positives and false negatives, were recorded.

## Results

Our analysis is based on a dataset of 32 recordings in 5 different brain regions in unrestrained rats during quiet rest and anaesthesia. During rest, recordings were obtained from the substantia nigra pars compacta (n = 8) and pars reticulata (n = 8), the deep mesencephalic nucleus (n = 3), the anterior pretectal nucleus (n = 6), and the zona incerta (n = 1). During anaesthesia, recordings were obtained from the anterior pretectal nucleus (n = 2) and the substantia nigra pars reticulata (n = 4). Neuronal activity was modified by iontophoretic application of glutamate or GABA to obtain spike trains with a wide dynamic range of firing frequency (n = 16). Figure 1A shows a typical recording of unit activity from a single cell and its response to iontophoretic application of glutamate. Under basal conditions the instantaneous firing rate was not stable but varied over short time intervals. Application of glutamate dramatically raised the firing rate of the neuron while reducing the mean height of the action potentials (between arrows). Three typical spike waveforms recorded from different brain regions are shown in Figure 1B.

### Similarity of Single-unit and Multi-unit Recordings

One of the premises of this study, that analysis of single-unit recordings is directly applicable to multi-unit recordings, relies on the assumption that extracted spike waveforms are similar in both cases. For comparison, we extracted spikes obtained from simultaneous multi-unit and intracellular recordings in anaesthetised rats from the Collaborative Research in Computational Neuroscience data-sharing website (crcns.org). The downloaded recordings were collected from the hippocampal CA1 region. The simultaneous intracellular recordings allowed unambiguous identification of single-unit activity in the multi-unit data (see Figure 1C). The spikes from 20 randomly selected CA1 neurons (Figure 1C, left panel) were similar to, but not exactly the same as, spikes from the single-unit recordings analysed for the current study (Figure 1C, right panel). The biggest differences were that the single-unit spikes were slightly shorter and had deeper hyperpolarisation after the peak.

For spike sorting, the waveform *per se* is not critical, but the difference between waveforms of different spiking cells determines spike-sorting success. Overall, the differences between all the single-unit waveforms were similar to the differences between all the multi-unit spikes, which themselves are typical of all multi-unit recordings (not shown). Conclusions we make regarding the difficulty of spike sorting using the single-unit waveforms are therefore also readily applicable to multi-unit recordings.

### Result 1: Spike-timing Variability

**Figure 2:**
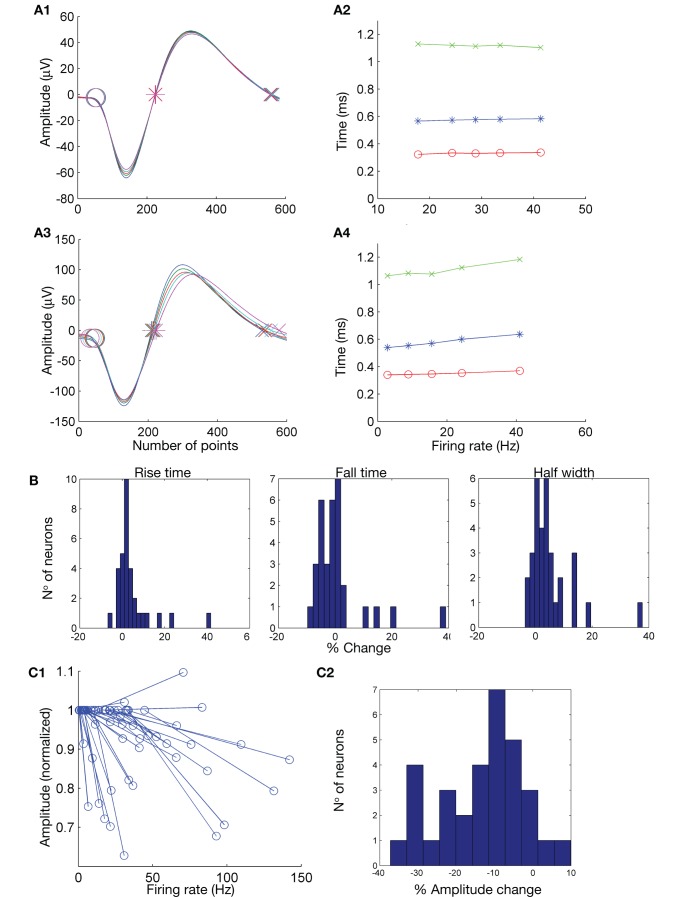
Action potential amplitude and shape vary with firing rate for the majority of neurons. A : Action potential spike-timing stability can vary with increased firing rate in certain neurons, as shown by two neurons (A1 and A3, stable and variable respectively) recorded from the substantia nigra pars reticulata in awake animals. Spikes were binned into five equally sized groups based on the firing rate of the neuron at the time each spike was emitted (see Methods for firing rate calculation method). The shapes of spikes in each bin were averaged, after which the spike start point (circles, left), first zero crossing (stars, left, corresponding approximately to the peak of the intracellular spike) and second zero crossing (crosses, left, corresponding to the approximate trough of the intracellular spike) were determined (see text for calculation methods). These points were then used to calculate spike rise time, fall time and half width (respectively blue, green and red lines on the graphs A2 and A4) for each spike bin. A1 and A2: No significant spike-timing change. A3 and A4: Significant spike-timing change. Significance was tested using an ANOVA of the five bins of each set of rise, fall and half-width times for each neuron. **B**: Rise-time, fall-time and half-width changes for all neurons. Mean changes were respectively +4.6±9.0% (p<0.007), +0.8±9.3% (p>0.64) and +5.2±7.9% (p<0.0008). Significance was tested with a standard one sample t-test. **C**: Spike amplitude decreased with increased firing rate. Spike amplitude at the lowest firing rate for each neuron was normalised to 1, after which the relative amplitude at the highest firing rate was plotted. 84% of cells displayed a significant amplitude decrease (C1). The distribution of maximum spike-amplitude variations (when a cell showed a transition from its lowest to its highest firing rate) across all cells reveals the majority of cells showed a clear spike-amplitude reduction (C2). The average amplitude change was –12.9±11.2% (p<10^−7^).

We define timing variability as changes in the zero-crossing times of spikes, irrespective of any amplitude change (*i.e.* amplitude change alone, without changes in zero-crossing times, is not a timing change). Although some neurons had stable and consistent spike timing, many showed minor spike-timing variability with increasing firing rate (Figure 2A). Cells with no significant timing changes (Figure 2A, top panels) showed stable spike rise time, fall time and half width as firing rate increased (Figure 2A2: blue  =  rise time; green  =  fall time; red  =  half width). Cells with significant timing changes (Figure 2A3–A4) generally showed increasing spike rise time, fall time and half width as firing rate increased. 47% of cells (15/32) had significant rise-time changes, with the mean change for these cells (± standard deviation) being +8.8±11.5% (p<0.02). Across all cells, the average rise-time change was +4.6±9.0% (p<0.007) with only 1 cell (3%) showing a significant decrease in rise time. The mean fall-time change was +0.8±9.3% (p>0.64), which was not significantly different from zero (*i.e.* even though 59% (19/32) of cells had significant individual fall-time changes, some increased and some decreased such that the average change for all cells together was not significantly different from zero – see Figure 2B showing histograms for percentage changes in spike rise times, fall times and half widths for all neurons). The mean absolute fall-time change was 5.3%. Overall, a rise-time change, fall-time change or both were observed for 75% (24/32) of the cells; 16% (5/32) had only a rise-time change, 28% (9/32) had only a fall-time change whereas 31% (10/32) had both.

Of all the recorded cells, 84% (27/32) had significant half-width changes with a mean change for these cells of +6.2±8.2% (p<0.0007). Only 3 cells (9%) showed a significant decrease in half width. Across all cells, the mean half-width change was +5.2±7.9% (p<0.0008).

To test for any effects of anaesthesia or pharmacology on spike timing, neurons were classified into the appropriate groups regardless of the brain region in which they were recorded. Anaesthesia and pharmacological manipulation had no significant effect on spike rise, fall and half-width times (Table 1). For comparison, the first two rows of Table 1 also summarise the results for changes in rise, fall and half-width times of all 32 neurons (for completeness, amplitude is also included – see next section for full amplitude change results). This absence of significant effects of anaesthesia and pharmacology indicates that it was valid to combine all neurons under all conditions for subsequent analyses in this study.

**Table 1:** Rise-time, fall-time, half-width and amplitude changes and significance levels for all cells, for awake *vs.* anaesthetised rats, and for control *vs.* pharmacologically manipulated conditions.

	Rise- time% change	Fall- time% change	Half- width% change	Amplitude% change
All cells mean ± std dev (n = 32)	4.6±9.0	0.8±9.3	5.2±7.9	–12.9±11.2
Significance level (p)	<0.007	>0.64	<0.0008	<10^−7^
Awake mean ± std dev (n = 11)	5.4±13.9	4.3±12.9	7.1±11.5	–14.2±14.1
Anaesthetised mean ± std dev (n = 5)	0.9±4.9	3.0±10.1	3.7±6.1	–8.4±7.5
Awake *vs*. Anaesthetised significance level (p)	0.501	0.841	0.545	0.410
No pharmacology (n = 27)	3.6±9.4	1.7±9.5	4.8±8.3	–12.1±11.2
Pharmacology (n = 16)	3.7±5.8	–2.4±4.9	3.8±4.3	–8.9±10.5
No pharmacology *vs*. Pharmacology (p)	0.972	0.121	0.674	0.360

Only rise time and half-width changes were significant for all cells (dark shading, one sample t-test). Significance for awake *vs.* anaesthetised and for no pharmacology vs. pharmacological conditions was tested using a two sample t-test. No significant differences were found for rise times, fall times and half-widths between awake and anaesthetised, or between pharmacology and no pharmacology conditions.

### Result 2: Spike Amplitude Variability

We evaluated spike amplitude variability based on Bonferroni-corrected t-tests, ANOVA tests and the slopes of lines of best fit on amplitude *vs.* firing rate graphs (see Methods). The majority of neurons (84%; 27/32) displayed significant amplitude reduction as the firing rate increased (see Figure 2C showing change in amplitude for each cell as the cell’s firing rate varied from minimum to maximum). Whereas the t-tests and slope tests gave the same significant differences for all cells, the ANOVA results differed from the former two tests for 1 cell, but still resulted in 81% (26/32) of cells showing significant amplitude reductions. Two cells (6%), both of which were in the substantia nigra pars reticulata, displayed a significant amplitude increase as the firing rate increased, although the increases were not large. Three cells (9%) presented with no significant amplitude change. There was a tendency for slower-firing cells, with maximum firing rates less than 50 Hz, to display larger amplitude reductions on average (see Figure 2C1). Across all 32 cells, the average amplitude change from the lowest to the highest of five firing-rate bins was -12.9±11.2% (p<10^−7^).

### Result 3: Effect of Noise on Spike Shape

**Figure 3:**
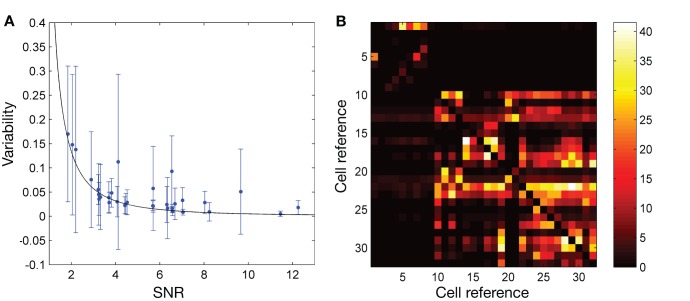
Spike variability increases dramatically at low SNRs and causes large misclassification errors. A : Spike-to-spike variability plotted against the SNR of the recording shows a rapid increase in variability at low SNR (A). Each data point indicates the average variability of each spike to the mean spike from that cell (see text for details). Error bars indicate one standard deviation. There was a slight general trend towards larger standard deviation in the variability at lower SNRs, although this was in addition to the effect of spike-timing changes which, for some cells, substantially increased the spike deviation even at higher SNRs. The fit curve shown is the fit line 1–√(1–1/*SNR* ^2^) (see text and Material S1). **B**: Percentage of spikes for each neuron that would be classified as coming from a different neuron if the two neurons were recorded simultaneously (and were recorded with similar SNR – that is, have similar amplitudes, so that amplitude could not be used to discriminate between the spikes). Cells 1 to 9 are cells with triphasic spikes (9 cells) whereas all others (23 cells) have biphasic spikes. Triphasic spikes are unlikely to be confused with biphasic spikes (the large, mostly dark regions at the top and left of the figure represent low misclassification rates for cells 1 to 9 when paired with all other cells). Cells 10 to 15 are anterior pretectal nucleus cells in awake (4 cells) and asleep (2 cells) animals, 16 to 27 are substantia nigra pars reticulata cells in awake (8 cells) and anaesthetised (4 cells) animals, 28 is a substantia nigra pars compacta cell in an awake animal, 29 is a zona incerta cell in an awake animal, and 30 to 32 are deep mesencephalic nucleus cells in awake animals.

Spikes became more affected by noise as the SNR of the spike recording decreased. This was evidenced by a rapid increase in spike-to-spike variability at low SNR (Figure 3A). Spike variability was calculated so that a variability of 0 indicated that every spike waveform from a given neuron was exactly the same (*i.e.* no variability) and that variability approached 1 as noise began dominating the waveforms (*i.e.* spike shapes became random; variability was calculated as (1– spike similarity), where spike similarity was the average, for all spikes from a cell, of the dot product of each spike with the mean spike shape for that cell; see Methods for details). As illustrated in Figure 3A, spike variability increased dramatically below an SNR of about 4, suggestive of a serious degradation in the quality of the recorded spikes. Conversely, there was little extra relative benefit in spike quality with a SNR greater than about 8. In many cases, a SNR between 4 and 8 may therefore be considered desirable in terms of the trade-off between cost or ease of recording and the recording quality.

Spike variability due to noise is related to SNR by the relation *variability*  = 1–√(1–1/*SNR* ^2^) (see Material S1). This relation is a good fit to the experimental data; the greater variability seen in the data for some cells is due to intrinsic spike-timing changes in those cells (see Result 1 above). Individual spikes are highly affected by noise, particularly at lower SNRs, to the extent that any single spike can appear more like a spike from a different neuron than a spike from the neuron that actually generated it. The likelihood of this confusion occurring can be calculated for this dataset by comparing all of the extracted spikes to the mean spike from each neuron (using the normalised dot product; see Methods). For any pair of neurons *a* and *b*, the proportion of spikes from neuron *a* which are more like the mean spike from neuron *b* than the mean spike of neuron *a* gives a theoretical minimum for the proportion of spikes from neuron *a* that will be misclassified when recording the two neurons together. Performing this calculation for all pairs of neurons in the current dataset (Figure 3B) yields a rate of misclassification ranging from 0% to 41.6%, with the mean misclassification across all biphasic spikes in the dataset (cells 10 to 32) being 8.3%. Note that cells with triphasic spikes (cells 1 to 9) are unlikely to be confused with cells with biphasic spikes (the large, mostly dark regions at the top and left of the figure represent low misclassification rates for cells 1 to 9 when paired with all other cells).

The cells in this study were selected for their recording stability and range of SNR levels, and selection was performed prior to any spike similarity analysis. It is therefore reasonable to expect that these cells are a representative sample of all cells in the investigated brain regions. When recording with a single electrode, a significant percentage of spikes can therefore be expected to be misclassified, even when recording from only two neurons at reasonably high SNRs and assuming perfect spike detection and sorting processes. The misclassifications arise for the simple reason that these spikes truly appear to originate from the opposing cell, due to noise-induced distortions of the spike shape. Crucially, these results are the theoretical minimum misclassification for the average case of all the recordings in the dataset used for this study. Although in some cases it would be possible to perform better classification (*e.g.* when recorded spikes are, by chance, very different), over many recordings the mean classification error will be at least as large as that reported. Thus, classification performance will be worse at least 50% of the time, even when recording from only two neurons simultaneously.

As the number of simultaneously recorded cells increases, the number of different ways that spikes can be misclassified also increases. Assuming a uniform distribution of spike shapes throughout the space of all possible shapes, then the correct classification percentage for any 3 simultaneously recorded cells from this dataset will, on average, be (100–8.3%)^2^ = 0.917^2^ = 84.1%, and in general the correctly classified proportion of spikes will be 0.917*^n^* ^–1^ where *n* is the number of recorded cells. For example, for *n* = 5, at most 71% of spikes will be classified correctly. The average SNR across all cells in this dataset is approximately 5; for lower SNRs, spike classification performance will be worse.

Given that the spike similarity measure used for the results above is invariant to spike amplitude (since the spike vectors are normalised – see Methods), one could suggest using spike amplitude as a discriminator to separate spikes into their respective functional units. We next tested the extent to which spike discrimination may be aided by the spike amplitude difference between neurons located at different distances from the recording electrode. Using reported neuronal densities, we modelled the combined amplitude and shape variations of spike waveforms expected from these neurons, assuming a random spatial distribution around the recording electrode. We chose a range of neuronal densities for non-layered brain areas based on the study by Oorschot [22].

### Result 4: Spike Misclassification as a Function of SNR

The previous result provided an estimate of the probability of misclassifying spikes based on spike shape alone. However, spike shape and amplitude information can be combined to determine the relationship between SNR and the probability of misclassifying each recorded spike. Given that the SNR at which a neuron is recorded is related to its distance from the *centre* of the electrode tip (so larger electrodes generally cause reductions in SNR), and given that we know the average densities at which neurons are distributed in the brain, it is possible to calculate, on average, how many neurons will be recordable (and the approximate SNRs of the recordings) for any given brain region [4]. Based on this calculation, the previous result can be applied to calculate the overall probability of misclassifying each recorded spike at any given SNR. We define SNR as RMS_signal_/RMS_noise_, where the signal comprises all extracted spikes and the noise comprises the remainder of the recording (see Methods for details). It is worth noting that the signal itself is contaminated by noise, and in our model we account for this by assuming that it is the same magnitude as baseline noise, and statistically independent of the signal. Note also that SNR will be significantly higher than reported here if it is calculated as Peak_signal_/RMS_noise_ (see Material S2 and Figure S3).

**Figure 4:**
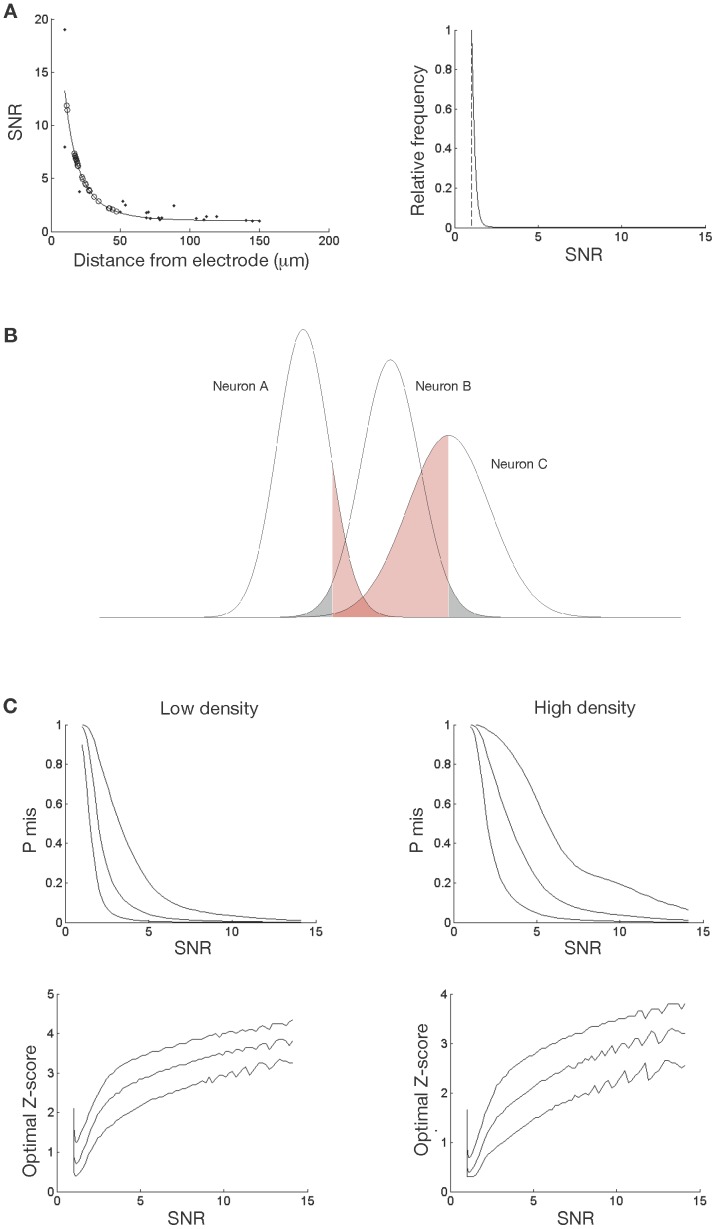
Most neurons will be recorded with an SNR of 2.5 or less; at these SNRs, the probability of misclassifying each spike approaches 1 (*i.e.* 100%) in some cases. **A**: The SNR follows the inverse square of distance. Left: Prediction of SNR *vs.* distance based on an assumed inverse square law of peak amplitude, average biphasic spike shape, and least squares-fitted to (assumed) peak amplitude *vs.* distance relationship (see text). Open circles show the SNRs of the recordings made for this study, fitted to the curve to estimate the distance to the recording electrode for each. Right: Marginal frequency distribution of spikes at lowest cell density, assuming 100% firing. For any randomly placed electrode, SNRs below 2.5 are most likely, and SNRs of 3 or more are unlikely to be recorded. Higher SNRs can potentially be obtained by strategic electrode placement (*e.g.* advancing the electrode towards a nearby cell to increase the SNR). **B**: Schematic illustration of estimation of spike misclassification probability, and minimisation of error using Z-score boundary (or cluster cut-off). The frequency distribution of spikes in amplitude–shape space for a hypothesised neuron (neuron B) is shown as the centre-most Gaussian curve. For a particular set of bounds around the mean of this distribution (in this case a Z-score of 2 or *μ±*2*σ*), a number of spikes from neuron B are incorrectly excluded from the cluster (false negatives in grey) whereas other spikes from surrounding neurons are included incorrectly (false positives in red). A Z-score limit for minimising the misclassification rate can be found for each distribution of neurons in amplitude–shape space, which varies according to the recording SNR and neural density in the particular brain region. C: Spike misclassification rates for single-wire recordings show that the probability of misclassifying any given spike approaches 1 at low SNRs. Top: Spike misclassification for lowest (left, 1.73×10^4^ neurons/mm^3^) and highest (right, 17.7×10^4^ neurons/mm^3^) cell densities (see text for details), assuming that 100% (top trace), 10% (middle trace) and 1% (bottom trace) of neurons fire. Bottom: Corresponding Z-score boundary which minimised the misclassification at each SNR. Note that the order of the traces is reversed (1%, 10% and 100% respectively from top to bottom).

In order to generalise this analysis we made a number of assumptions. The first of these is that spike amplitude varies as the inverse square of the distance of the neuron from the recording electrode (*i.e.* 1/*r* ^2^ where *r* is the electrode distance), following experimental observation and the approximation of a neuron as a dipole current source, which holds up to very short distances [3]. For completeness, we also tested 1/*r* and linear relationships of amplitude to distance. All relationship models were fitted to data obtained from simultaneous intra- and extra-cellular recordings [26], where the distance to the extra-cellular electrode from the recorded neuron was known, allowing estimation of the relationship between spike amplitude and recording distance (see Figure S4, Table S1 and Material S3 for details). Results for the 1/*r* assumption differ only slightly from 1/*r* ^2^; results for the linear assumption give lower SNRs and higher misclassification rates than observed in practice, providing further evidence for the invalidity of this model. Figure 4A, left panel, shows recording SNR as a function of distance of the recorded neuron from the electrode assuming the inverse square model. The maximum SNR at which a neuron can be recorded, when the neural membrane is as close as possible to the electrode tip, is approximately 16 (assuming an electrode diameter of about 20 µm, and that SNR is calculated as RMS_signal_/RMS_noise_), and drops rapidly as the distance to the electrode increases [26]. Due to tissue damage, only neurons from the volume beyond the electrode tip are recorded.

Our second assumption is that neural density ranges from 1.73 to 17.7×10^4^ neurons/mm^3^ for the regions we are considering, giving a total number of neurons within the recordable volume in the range of approximately 122 to 1250 [22], [27]. Depending on the brain region being recorded, not all of these neurons can be assumed to spike on any given occasion – we present results for 100%, 10% and 1% firing. Finally, we assume that spike-shape variability is due to noise only (since in practice, intrinsic spike shape variability is small compared to noise, even at higher SNRs), amplitude variation is independent of spike-shape, and spike ‘clusters’ in amplitude–shape space are circularly symmetric Gaussian (for Gaussian noise distribution see Figure S2). Given these assumptions, it is possible to calculate how many neurons on average can be recorded at each SNR (see Figure 4A, right panel, showing that the majority of recorded neurons will have a very low SNR). For randomly placed electrodes, more than 99% of recorded spikes will have a SNR below 3.

Based on our assumptions, within the spike amplitude–shape space, spikes from each recorded neuron will form clusters, and these clusters will overlap to varying extents depending on the SNR and the neuron density. We estimate the probability of misclassification *P_mis_* of spikes from a neuron, accounting for both false positives (spikes from other neurons) and false negatives (missing spikes), as:
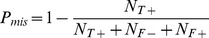
(2)where *N* is the number of spikes, *T+* indicates true positives, *F–* indicates false negatives, and *F+* indicates false positives. Figure 4B shows how overlapping spike clusters (neurons A and C) for a recorded neuron (neuron B) cause both false positive errors (red shading) and false negative errors (grey shading) which depend on the extent of overlap of the clusters as well as where the cluster boundary is drawn. Through numerical simulations, it is possible to find the Z-score boundary which minimises this error. We ran simulations that sampled mean spike shapes and spike shape variability directly from the biphasic cells recorded for this study. Once the Z-score limit was determined in this way, the minimum possible misclassification error, which is equivalent to the minimum probability of misclassifying each recorded spike, as a function of recording SNR in different brain regions, could also be estimated. The probability of misclassifying any given spike approaches 1 at low SNRs. Figure 4C shows the resulting spike misclassification rates (top) for lowest (left) and highest (right) cell densities, assuming that 100% (top trace), 10% (middle trace) and 1% (bottom trace) of neurons fire. Figure 4C (bottom) also shows the corresponding optimal Z-score limits for minimising misclassification at any given SNR. From these graphs, the minimum SNR required to obtain a given misclassification rate can be determined (see Table S6 for common examples). Similar results for the linear model of SNR to recording distance are given in Figures S5, S6 and Table S2, and for the inverse model in Figures S7, S8 and Tables S3 and S4. For completeness, results in Figure 4 are reproduced in Figures S9, S10 and Table S5.

We define an arbitrary ‘acceptable’ misclassification probability *P_mis_*< = 0.1; that is, misclassification of at most 10% of spikes constitutes an acceptable performance level. From these results, it is clear that acceptable spike discrimination at practical SNRs can be achieved, under favourable conditions, in brain regions with low neural density provided that 10% or less of all neurons fire in any given recording. However for high density brain regions, where neural density is increased more than ten times, the proportion of firing cells understandably must be around 1% in order to achieve acceptable spike classification at typical SNRs. If 10% of neurons in a high density brain region are firing, then the SNR must be greater than 6 to achieve 90% classification success; at an SNR of 4, at least 40% of spikes would be misclassified. This poor performance is attributable to 1) the similarity of biphasic spike shapes in general, 2) the large number of recordable neurons in high density brain regions, and 3) the large spike shape variability due to noise at typical SNRs.

### Result 5: Spike Classification Examples

**Figure 5:**
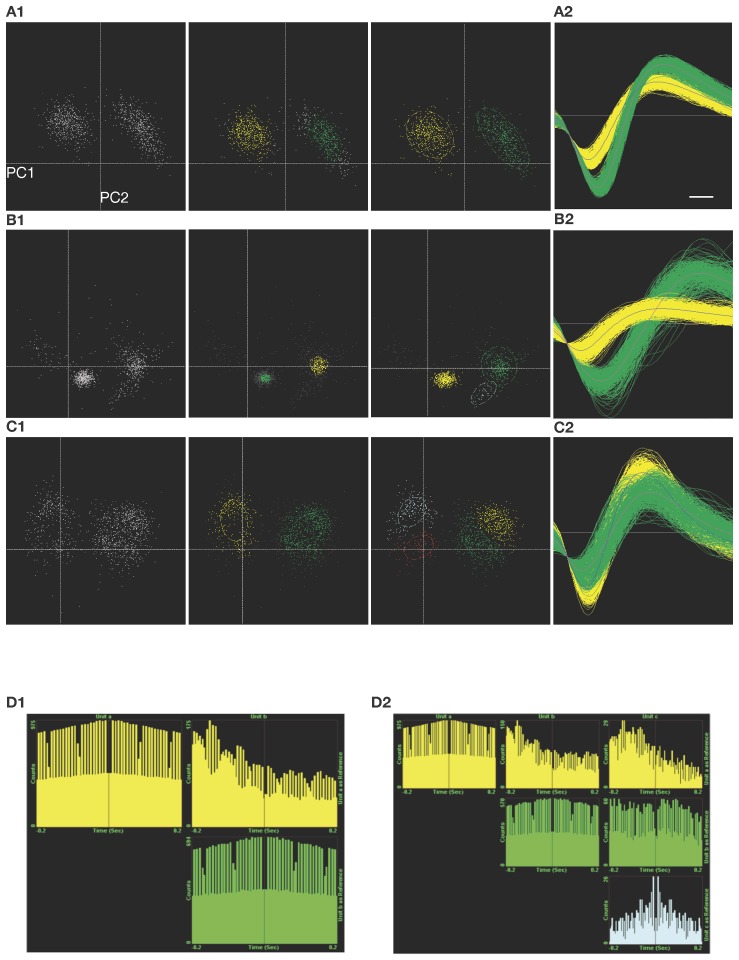
Spike sorting examples show common difficulties of separating spikes from different neurons at all but very high SNRs. **A**: Hybrid trace 1 was easily separated into two clusters corresponding to spikes from the two cells (the axes for all hybrid traces in this figure are the first and second principal components of spike shape). 5A1 Left: Initial unsorted clusters. Middle: Spike classification using K-means scan clustering with default parameters, which detected the two clusters but failed to correctly allocate all spike waveforms. Right: Spike classification using valley-seeking clustering, which correctly allocated almost all spike waveforms. 5A2: Sorted waveforms. **B**: Hybrid trace 2 was more difficult to separate into the two clusters corresponding to spikes from the two cells. 5B1 Left: Initial unsorted clusters showing large spread of one cluster and significant noise. Middle: Spike classification using K-means scan clustering with default parameters, which detected the two major clusters but failed to correctly allocate all spike waveforms. Right: Spike classification using valley-seeking clustering, which incorrectly detected three spike clusters. 5B2: Ideally sorted waveforms (not achieved by the sorting). **C**: Hybrid trace 3 was difficult to separate into the two clusters corresponding to spikes from the two cells. 5C1 Left: Initial unsorted clusters; spikes around and to the left of the midline are noise whereas spikes to the right of the midline are the spikes from the two cells. Middle: Spike classification using K-means scan clustering with default parameters, which detected a noise cluster and a cluster combining the two cells. Right: Spike classification using valley-seeking clustering, which detected the two spike clusters as well as two noise clusters. 5C2: Ideally sorted waveforms. **D**: Systematic classification errors can cause false interpretations of data that are very difficult to detect. 5D1: Auto-correlograms and the cross-correlogram for hybrid trace 2 with two correctly identified units (auto-correlograms are on the main diagonal). 5D2: Auto- and cross-correlograms for hybrid trace 2 with three falsely identified units, showing that the extraneous third unit appears to have different firing characteristics (the auto-correlogram at bottom right) and different interactions with the other identified spike sources (cross-correlograms off the main diagonal). Based on this result, the erroneous identification of three distinct neurons in this recording would appear to be well justified.

To test the above predictions using a common spike-sorting system, hybrid spike traces were created by concatenating spikes from several cells into a continuous simulated recording (each hybrid recording contained two cells; 500 spikes from one cell were appended together, followed by 500 spikes from another cell; see Methods for details). Hybrid trace 1 contained two cells with similar spike shapes (spike similarity = 0.992), but recorded at high and distinct SNRs (respectively 6.3 and 11.4) giving them distinctly different amplitudes. Using K-means scan sorting with the default cluster size Z-score of 1.2 standard deviations (Plexon Offline Sorter) two distinct clusters were identified (by 4 of 5 cluster measures), corresponding to the spikes from the two cells (see Figure 5A1, left and middle). The misclassification rates were 8.8% and 26% respectively. However, most of the misclassifications were false negatives (spikes omitted from the clusters); by increasing the cluster Z-score cut-off to 2.0, misclassification was reduced to 1.2% and 2.6% (19 spikes of the 1000 were misclassified in total, of which 16 were still false negatives, or omissions, and 3 were false positives, or attribution of the spike to the wrong cell; data not shown). The use of valley-seeking sorting with default parameters (Plexon Offline Sorter) also identified two distinct clusters corresponding to the spikes from the two cells (see Figure 5A1, right). Figure 5A2 shows the actual spike waveforms. Misclassification was 0.8% for each cell (8 spikes of the 1000 were misclassified in total, of which 6 were false negatives and 2 were false positives). These results agree well with those predicted (Figure 4C) and illustrate that, with high recording SNR, good spike classification can be achieved.

Hybrid trace 2 contained two cells recorded at moderate SNRs (5.8 and 3.7) with moderately different spike shapes (spike similarity = 0.958). Using K-means scan sorting with the default cluster size Z-score of 1.2 standard deviations (Plexon Offline Sorter) two distinct clusters were identified (by 2 of 5 cluster measures), corresponding to the spikes from the two cells (see Figure 5B1, left and middle). The misclassification rates were 63% and 60% respectively, although most of the misclassifications were false negatives; by increasing the cluster Z-score cut-off to 2.0, misclassification was reduced to 6.6% and 18% respectively (123 spikes of the 1000 were misclassified in total, of which 96 were false negatives and 27 were false positives; data not shown). Valley-seeking sorting with default parameters identified *three* distinct clusters, where spikes from one of the cells were correctly identified but spikes from the other cell were split into two separate clusters (see Figure 5B1, right). If spikes in the smaller of these two clusters were treated as false negatives, then misclassification in this case is 1.6% for the correctly identified unit and 26% for the split unit (all 137 misclassified spikes across both cells were false negatives in this case). These results agree well with predictions (Figure 4C) and illustrate the difficulty in distinguishing spikes from as few as two cells when recorded at typically ‘good’ SNRs.

Hybrid trace 3 contained two low SNR cells (3.3 and 2.4) with moderately dissimilar spike shapes (spike similarity = 0.929). Using K-means scan sorting with the default cluster size Z-score of 1.2 standard deviations, two distinct clusters were identified (by 3 of 5 cluster measures) but these clusters did not correspond to the spikes from the two cells; instead one cluster incorporated a large number of spikes from both cells and the other clearly comprised only noise (see Figure 5C1, left and middle). The noise arose from spikes crossing threshold much earlier than the actual spike peak, or spikes so distorted by noise that they were unrecognisable. The misclassification rate for the single spike cluster was 53% (139 false negatives and 263 false positives). Valley-seeking sorting with default parameters identified four distinct clusters, two of which were clearly noise whereas the others corresponded to the spikes from the two cells (see Figure 5C1, right). Misclassification was 27% and 43% (across the two cells, 305 misclassified spikes were false negatives and 75 were false positives). This is a favourable result for a low SNR recording, and approaches the predicted best value (Figure 4C).

Systematic classification errors can have a large deleterious effect on spike sorting and subsequent interpretation of results. In hybrid trace 2 (Figure 5B) an extraneous third spike source could, depending on the spike-sorting technique, be extracted from the recording. Because this extraneous spike source generally comprised the smaller spikes emitted by the real cell in question, and because those smaller spikes tended to be emitted during long spike bursts from that cell, there was a systematic distortion of spike times that caused the extraneous spike source to look like a different cell when judged based on the spike time auto- and cross-correlograms. Figure 5D1 shows the correct correlograms; Figure 5D2 shows the correlograms with the extraneous cell, which appears to have significantly different firing characteristics even though all the spikes originate from one of the original cells. It is concerning that such a significant error is so easy to make, and that there should be no substantial evidence that an error has occurred.

To summarise, the best classification obtained for each hybrid trace was: 1) high SNR case –0.8% misclassification for both cells; 2) moderate SNR case – an average of 12% misclassification across the two cells; 3) low SNR case – an average of 35% misclassification across the two cells. Note that different methods obtained the best classification in each case, classification errors can vary widely depending on the method used, and in practice it is impossible to determine which method is returning the best result in each case. In addition, the misclassification rates for hybrid traces 2 and 3 are not necessarily indicative of overall misclassification rates for recordings made at these moderate and low SNRs. These hybrid traces each contained spikes from only two cells, whereas recordings made at low SNRs are likely to contain spikes from many more neurons (see Figure 4A) which will further increase spike misclassifications in practice. Finally, systematic classification errors can introduce large erroneous biases in and misinterpretations of recording data. Therefore, these three hybrid trace examples should be considered the expected best possible result for recordings at each of these SNRs, and generally classification errors will be higher, and indeed much higher in some cases.

## Discussion

Here we studied action potential waveform variability and the possibility of obtaining accurate spike sorting from single-channel recordings. Our analysis confirms the previous observation that spike amplitude decreases and spike duration increases with increasing firing rate [11], [28], [29]. We used local pharmacology to control firing rate and obtain sufficient spikes to assess waveform variability across a large dynamic range (Figure S1) within the observed physiological limits (from baseline to maximum firing frequency). The frequency-modulated amplitude and spike timing observed during pharmacological manipulations were in agreement with those obtained from action potentials extracted at a baseline firing rate. During recordings, great care was taken to ensure that single units were isolated, with the consistent results obtained for spike amplitude and duration changes across a very broad range of SNRs indicating that, in general, the isolation was successful. In a handful of recordings, some very small spikes, presumably from different neurons, were visible, but these were easily separated based on amplitude alone and were excluded from analysis by appropriate setting of the spike detection threshold.

Anaesthesia had no effect on spike timing or frequency-modulated amplitude. In our experimental conditions two major factors related to anaesthesia had the potential to influence the action potential waveforms. First, temperature is known to alter spike waveforms [30]–[32]; however keeping the body temperature constant seemed to be sufficient to obtain a brain temperature that did not affect action potentials. Second, spike shape can be influenced by the actions of neurotransmitters [33], [34], a function that is highly modified by anaesthesia [18]. However the fact that the extracellular action potential waveform and its frequency modulation were not significantly influenced by anaesthesia validates the method of using waveform to identify, in anaesthetised *in vivo* preparations, neurons previously recorded in the same animal in behavioural experiments [10]. In unrestrained, non-anaesthetised animals, physiological temperature fluctuations [35] probably play a role in the action potential waveform variations we observed.

We have demonstrated that spike amplitude and timing can vary substantially from spike to spike but that the overall waveform variability is also greatly affected by the SNR at which spikes are recorded; waveform variability increases dramatically at a low SNR. Although it is possible to record and extract action potentials from a neural signal with an SNR as low as 2 or 3, the large variability in waveform shape due to noise means that many of the action potentials will be indistinguishable from the waveforms generated by other neurons. For instance, for one of the neurons recorded at low SNR in this study, more than 40% of its spikes could be confused with spikes generated by another neuron even if recording from only those two neurons. Clearly, as more neurons are added to the recording, the misclassification rate only increases. Separating multiple units at very low SNR is therefore not viable.

A dramatic improvement in spike separability is seen with an SNR ≥4. When recording two neurons, error rates drop to a mean of 8.3% for a mean SNR around 5. However, even with a large SNR, the rate of correct classification decreases as more neurons are recorded. As a general approximation, the correct classification percentage for *n* simultaneously recorded neurons is equal to 100*(0.917)^n-1^ for a SNR of approximately 5. Naturally, classification performance degrades as the SNR reduces and improves as the SNR increases. In addition, the probability of recording two neurons at a high SNR is very low (equal to the probability of the single electrode being very close to two neurons simultaneously); conversely, the probability of recording more than two neurons (perhaps many more) at a low SNR is quite high. Therefore a neuron recorded at a very high SNR will usually be distinct as it is likely to be the only neuron in the recording with such a high SNR (*i.e. n* = 1). However, a neuron recorded at a moderate SNR will often have many similar companions (*n* = large). In practice then, a neuron recorded at a high SNR will usually be distinctly different and clearly separable, whereas neurons recorded at moderate SNRs will often be subject to large classification errors.

In addition to single-channel spike classification using spike waveforms, a technique based on the neuron’s refractory period is commonly used to detect contamination of one spike train by action potentials from another neuron. The autocorrelation of the spike train from one neuron should show a distinct shape, with no spikes detected following the zero time point for the duration of the refractory period. Nevertheless the value of this method is limited. First, it facilitates detection of classification errors but is not informative in terms of the potential origin of the spikes contaminating the spike train. Thus, while in some cases the autocorrelation may indicate that an error in spike sorting has been made, it provides no information on how to correctly reclassify the spikes. Second, it is less efficient for cells with a low firing rate. Concluding from a spike train autocorrelation that action potentials have a unique point of origin requires the number of detected spikes to be sufficiently large, such that some of these appear within the refractory period. If the number of action potentials is low, it might preclude drawing a conclusion regarding the accuracy of the spike sorting based on the autocorrelation. For example, with 2 neurons firing at 5Hz, only 1.5% of the spikes will appear within the 3 ms refractory period (assuming independent Poisson spike distributions). If there are interactions between the neurons in question (non-Poisson spike distributions) then it is possible that no violation of refractory periods will be seen, even given high firing rates and/or long recording times.

The decrease in spike amplitude with increased firing rate, combined with amplitude variability due to noise, implies that, in many cases, spike amplitude provides negligible information in the discrimination of spikes (*i.e.* two spikes with the same shape but different amplitude might have the same origin because of the frequency modulation of the spike amplitude and noise-induced amplitude variability). Conversely, when recording two or more cells simultaneously using a single electrode, even if the cells’ spikes have very different mean amplitudes, noise and frequency-modulated amplitude may lead to some spikes from the two cells having similar amplitudes (see Material S4 for derivation).

Typically, action potentials recorded extracellularly originate from the cell soma, this being the strongest current source [1], [2]. The electrical signal from the soma then tends to travel mostly isotropically (uniformly) through the brain [13], [36], resulting in the stereotypical spike shapes which dominate neural recordings. However there are at least two ways in which altered spike shapes may appear. Discontinuity in the homogeneity of the neuropil (e.g. fibre tracts or cell clusters) may disrupt this isotropy and alter the spike waveform. It is also possible that in certain cases the action potential is recorded from neuronal processes away from the soma, but in these cases such recordings have to be made very close to the source because of the low amplitude of the current [2], [37]. Although this non-somatic source may produce action potential waveforms radically different from the stereotypical spike shape [2], [37], [38], these cases are rare due to the proximity to the neuropil required. The similarity of spike shapes in general, and the poor assistance offered by spike amplitude together make the spike-sorting problem particularly difficult to solve.

When waveforms from two neurons are combined into one hybrid spike train and tested with spike sorting, the spike-to-spike variability induces large error rates related to spike shape similarity and recording SNR as well as spike-sorting parameters. We have shown that for recordings at high SNR, the allowable unit cluster size should be increased (equivalently, the outlier threshold after which spikes are rejected from the closest cluster should be extended, so that fewer spikes are rejected). Small clusters (equivalently low outlier thresholds) favour low numbers of false positive spike detection errors, but suffer from high numbers of false negative detection errors for higher SNR recordings.

To summarise, physiological changes (frequency-modulated spike amplitude and timing changes) and technical limitations (SNR) reduce the accuracy of single-channel spike classification for non-layered brain regions. Nevertheless, for low density brain regions where 10% or less of neurons are firing, good spike classification (defined as 90% or better) can be obtained with SNR = 4 or more. For SNR>7, nearly perfect classification can be achieved. For high density regions, however, SNR>6 is required for good classification; SNR = 4 will achieve at best 60% correct classification even if only 10% of neurons are assumed to be firing. In almost all circumstances, SNR<4 will result in an unacceptably high spike classification error. Recall that SNR in this study was calculated as RMS_signal_/RMS_noise_; SNR limits will generally be at least 50% higher if SNR is calculated as Peak_signal_/RMS_noise_ (see Material S2).

This result has significant implications for studies that rely on identification of individual units in multi-unit recordings. Two distinct types of classification error exist – firstly, random classification errors caused by noise and, secondly, systematic classification errors caused by intrinsic changes in spike shape and amplitude or a multitude of other time-varying effects (electrode movement or deterioration, inflammation due to tissue damage, non-stationary noise, temperature differences and countless other potential environmental and physiological changes). Random spike classification errors due to noise will make statistical significance more difficult to demonstrate, and the difficulty will increase as SNR decreases. For example, correlations between the firing of individual neurons (implying involvement of the neurons in a common underlying circuit) or between neural activity and stimuli or behaviour, will be obscured, requiring more data to reach a comparable significance level. Similarly, as spike-to-spike variance increases, spike clusters become more diffuse and may overlap significantly, making it harder to determine both the number of clusters present and to which cluster any given spike belongs (*e.g.* see Figure 5C).

Systematic classification errors, rather than purely random errors, can have other highly deleterious effects. For example, intrinsic spike amplitude and shape changes, which often occur systematically based on firing rate, can be mistaken as evidence for the existence of multiple neurons in a recording. Because the errors are systematic, such as the generation of low-amplitude spikes at high firing rates and during spike bursts, these erroneously identified extra neurons can appear to have very different firing properties and can even be taken as strong evidence for a presumed neural circuit; however in these cases the circuit would be completely fictitious (*e.g.* see Figure 5B and 5D). Systematic classification errors, rather than just obscuring the significance of results, can cause the appearance of results that are entirely fallacious.

At low recording SNRs where noise is a substantial proportion of the signal, random errors dominate; systematic errors may be present but they are obscured by the large spike variance and diffuse spread of the spike clusters. The use of multi-wire recording techniques, such as tetrodes, can significantly reduce the incidence of random spike classification errors, as the probability of noise corrupting all recording channels simultaneously is substantially lower than noise corrupting only one channel. Tetrodes, however, have the disadvantages of being more complex to use, creating more data to manage and potentially increasing tissue damage, as a result of which single electrodes continue to be used in many experiments. Interestingly, tetrodes may be of little benefit when facing systematic classification errors, since a spike shape which is systematically changing on one channel is also likely to be systematically changing on other channels. Therefore systematic classification errors caused, for example, by correlations of spike amplitude or spike shape with firing rate, as presented in this paper, may also commonly cause spike misclassifications for tetrode recordings. Systematic errors become significant at moderate to high SNRs (Figure 5B and 5D).

Electrodes used in behavioural experiments usually have a relatively large diameter, resulting in reduced SNR (since, as for all inverse and inverse-square laws, the SNR at which a neuron is recorded decreases with increasing distance from the *centre* of the electrode tip). In practice, SNRs above 5 are difficult to obtain with these electrodes, which severely limits the number of different spike shapes (individual neurons) that can be successfully classified. Different brain regions have widely varying neural densities as well as widely varying firing probabilities. For brain regions with high neural density and/or high firing probability, such as the cortex, hippocampus, thalamus, and substantia nigra pars reticulata, our analysis indicates that separation of spikes using single-electrode recordings is likely to result in large classification errors, even at what is typically regarded as a ‘good’ SNR (*e.g.* ∼4). Even in low density brain regions, spike classification errors will be high if more than 10% of neurons are firing or if the SNR is much less than 4. In addition, in those cases where a high SNR is obtained, the necessary proximity of the electrode to the cell means that even a small shift in electrode position will produce a large change in the relative distance to the cell, resulting in a large amplitude change, which may then cause the cell to be reclassified as a new spike source. Based on our simulated and experimental results, classification errors will increase rapidly for the SNRs typically used in experiments with awake behaving subjects unless the recording SNR is at least 4 (or close to 10 if SNR is calculated as spike peak amplitude divided by RMS_noise_) and both neural density and firing probability are low. When recording with single electrodes it is therefore vital to consider both the SNRs of the recordings being obtained and the neural density of the brain regions being recorded, bearing in mind that the latter vary between species [39]. Identification of distinct single units and interpretation of their driven and correlated activity need to be carefully considered in light of these constraints.

## Supporting Information

Figure S1 **Action potential waveform amplitude to firing frequency variation for one neuron, recorded in unrestrained non-anaesthetised conditions.** Blue circles and red dots represent action potentials recorded during baseline activity or glutamate iontophoresis respectively. Notice that glutamate iontophoresis reduces the likelihood of activity at low firing frequencies but does not alter the waveform amplitude or cause significant activity at firing frequencies higher than baseline.
(TIFF)

Figure S2 **Noise distribution for the first 2 seconds of recording in the substantia nigra pars reticulata in an awake unrestrained rat with Gaussian fitted.** A Kolmogorov-Smirnov test supports a Gaussian noise distribution (p = 0.5418).
(TIFF)

Figure S3 **Graphic representation of SNR and SNR_a_ relation; see text for details.**
(TIFF)

Figure S4
**Model fits to extracellular spike amplitude data, and predicted 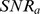 and 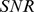 (S15).** For consistency, all SNR curves start at 10 µm (assumed radial distance of cell at 600 µV). The extracellular spike amplitude (left column) refers to the average maximum deflection from baseline. The RMS noise (left column) is shown as dashed lines. The 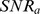, 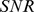 and estimated distances are shown for data used in this work for the non-linear models (circles). The peak deflection of all mean spike shapes were between 50.4% and 68.6% of the peak-to-peak amplitude. Data are courtesy of the Buzsaki group from the Collaborative Research in Computational Neuroscience data-sharing website (crcns.org).(TIFF)

Figure S5 **For the linear model, expected probability of misclassification,** ***P_mis_*** **, is unacceptably high for almost any attainable recording SNR.** Left: *P_mis_* for low density (top) and high density (bottom) brain regions as a function of recording SNR. Top trace: 100% of neurons firing; middle trace: 10% firing; bottom trace: 1% firing. Right: Optimal cluster Z-score sizes for each case (note that the top-to-bottom order is reversed; that is, the traces represent 1%, 10% then 100% of neurons firing). At low firing rate and low density, the lower average misclassification rate is partly due to the linear drop in spike amplitude, which means that there are relatively few cells with low SNR. On average there is only approximately one cell firing (4 cells per 50 µm radius hemisphere  = 108 cells per 150 µm hemisphere, which at 1% firing is about 1 cell). For 90% success rate, an SNR of 2.8 is sufficient in this case. Notice, however, that the highest attainable SNR for the linear model is about 3, which does not fit with experimental observations.
(TIFF)

Figure S6 **Expected relative frequency of the SNR of neural recordings for randomly placed electrodes, for the linear model.**
(TIFF)

Figure S7 **For the inverse model, expected probability of misclassification,** ***P_mis_*** **, approaches 1 at low recording SNRs.** Left: *P_mis_* for low density (top) and high density (bottom) brain regions as a function of recording SNR. Top trace: 100% of neurons firing; middle trace: 10% firing; bottom trace: 1% firing. Right: Optimal cluster Z-score sizes for each case (note that the top-to-bottom order is reversed; that is, the traces represent 1%, 10% then 100% of neurons firing).
(TIFF)

Figure S8 **Expected relative frequency of the SNR of neural recordings for randomly placed electrodes, for the inverse model.**
(TIFF)

Figure S9 **For the inverse square model, expected probability of misclassification,** ***P_mis_*** **, approaches 1 at low recording SNRs.** Left: *P_mis_* for low density (top) and high density (bottom) brain regions as a function of recording SNR. Top trace: 100% of neurons firing; middle trace: 10% firing; bottom trace: 1% firing. Right: Optimal cluster Z-score sizes for each case (note that the top-to-bottom order is reversed; that is, the traces represent 1%, 10% then 100% of neurons firing).
(TIFF)

Figure S10 **Expected relative frequency of the SNR of neural recordings for randomly placed electrodes, for the inverse square model.**
(TIFF)

Material S1 **Relating spike-shape variability to SNR.**
(DOC)

Material S2 **Modelling SNR in terms of spike amplitude variation and spike shape.**
(DOC)

Material S3 **Relationship between SNR and distance from electrode tip.**
(DOC)

Material S4 **Unified probability of spike misclassification.**
(DOC)

Table S1 **Fit parameters and the corrected Akaike Information Criterion for each model.**
(TIFF)

Table S2 **Percentage of cells above a given SNR assuming random cell and electrode positions, for the linear model.**
(TIFF)

Table S3 **Percentage of cells above a given SNR assuming random cell and electrode positions, for the inverse model.**
(TIFF)

Table S4
**Minimum SNR required to achieve a given success rate defined as 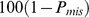, for the inverse model.**
(TIFF)

Table S5 **Percentage of cells above a given SNR assuming random cell and electrode positions, for the inverse square model.**
(TIFF)

Table S6
**Minimum SNR required to achieve a given success rate defined as 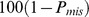, for the inverse square model.**
(TIFF)

## References

[1] Rall W (1962). Electrophysiology of a dendritic neuron model.. Biophys J.

[2] Gold C, Henze DA, Koch C, Buzsaki G (2006). On the origin of the extracellular action potential waveform: A modeling study.. J Neurophysiol.

[3] Mechler F, Victor JD (2012). Dipole characterization of single neurons from their extracellular action potentials.. J Comput Neurosci.

[4] Buzsaki G (2004). Large-scale recording of neuronal ensembles.. Nat Neurosci.

[5] Lewicki MS (1998). A review of methods for spike sorting: the detection and classification of neural action potentials.. Network.

[6] Takekawa T, Isomura Y, Fukai T (2010). Accurate spike sorting for multi-unit recordings.. Eur J Neurosci.

[7] Gray CM, Maldonado PE, Wilson M, McNaughton B (1995). Tetrodes markedly improve the reliability and yield of multiple single-unit isolation from multi-unit recordings in cat striate cortex.. J Neurosci Methods.

[8] Cromwell HC, Anstrom K, Azarov A, Woodward DJ (2005). Auditory inhibitory gating in the amygdala: single-unit analysis in the behaving rat.. Brain Res.

[9] Tye KM, Janak PH (2007). Amygdala neurons differentially encode motivation and reinforcement.. J Neurosci.

[10] Herry C, Ciocchi S, Senn V, Demmou L, Muller C (2008). Switching on and off fear by distinct neuronal circuits.. Nature.

[11] Fee MS, Mitra PP, Kleinfeld D (1996). Variability of extracellular spike waveforms of cortical neurons.. J Neurophysiol.

[12] Quirk MC, Wilson MA (1999). Interaction between spike waveform classification and temporal sequence detection.. J Neurosci Methods.

[13] Yedlin M, Kwan H, Murphy JT, Nguyen-Huu H, Wong YC (1974). Electrical conductivity in cat cerebellar cortex.. Exp Neurol.

[14] Rebec GV, Langley PE, Pierce RC, Wang Z, Heidenreich BA (1993). A simple micromanipulator for multiple uses in freely moving rats: electrophysiology, voltammetry, and simultaneous intracerebral infusions.. J Neurosci Methods.

[15] Towe AL, Harding GW (1970). Extracellular microelectrode sampling bias.. Exp Neurol.

[16] Humphrey DS, Schmidt EM, Boulton AA, Baker GB, Vanderwolf CH (1990). Extracellular single-unit recording methods..

[17] Windels F, Kiyatkin EA (2003). Modulatory action of acetylcholine on striatal neurons: microiontophoretic study in awake, unrestrained rats.. Eur J Neurosci.

[18] Windels F, Kiyatkin EA (2004). GABA, not glutamate, controls the activity of substantia nigra reticulata neurons in awake, unrestrained rats.. J Neurosci.

[19] Grofova I, Deniau JM, Kitai ST (1982). Morphology of the substantia nigra pars reticulata projection neurons intracellularly labeled with HRP.. J Comp Neurol.

[20] Ottersen OP, Storm-Mathisen J (1984). GABA-containing neurons in the thalamus and pretectum of the rodent. An immunocytochemical study.. Anat Embryol (Berl).

[21] Cucchiaro JB, Bickford ME, Sherman SM (1991). A GABAergic projection from the pretectum to the dorsal lateral geniculate nucleus in the cat.. Neuroscience.

[22] Oorschot DE (1996). Total number of neurons in the neostriatal, pallidal, subthalamic, and substantia nigral nuclei of the rat basal ganglia: a stereological study using the cavalieri and optical disector methods.. J Comp Neurol.

[23] Nair-Roberts RG, Chatelain-Badie SD, Benson E, White-Cooper H, Bolam JP (2008). Stereological estimates of dopaminergic, GABAergic and glutamatergic neurons in the ventral tegmental area, substantia nigra and retrorubral field in the rat.. Neuroscience.

[24] Windels F, Kiyatkin EA (2006). General anesthesia as a factor affecting impulse activity and neuronal responses to putative neurotransmitters.. Brain Res.

[25] Paxinos G, Watson C (1986). The rat brain in stereotaxic coordinates.. San Diego, California, USA: Academic Press Inc.

[26] Henze DA, Borhegyi Z, Csicsvari J, Mamiya A, Harris KD (2000). Intracellular features predicted by extracellular recordings in the hippocampus in vivo.. J Neurophysiol.

[27] Zhang R, Oorschot DE (2006). Total number of neurons in the habenular nuclei of the rat epithalamus: a stereological study.. J Anat.

[28] McCormick DA, Connors BW, Lighthall JW, Prince DA (1985). Comparative electrophysiology of pyramidal and sparsely spiny stellate neurons of the neocortex.. J Neurophysiol.

[29] Bourque CW, Renaud LP (1985). Activity dependence of action potential duration in rat supraoptic neurosecretory neurones recorded in vitro.. J Physiol.

[30] Thompson SM, Masukawa LM, Prince DA (1985). Temperature dependence of intrinsic membrane properties and synaptic potentials in hippocampal CA1 neurons in vitro.. J Neurosci.

[31] Guatteo E, Chung KK, Bowala TK, Bernardi G, Mercuri NB (2005). Temperature sensitivity of dopaminergic neurons of the substantia nigra pars compacta: involvement of transient receptor potential channels.. J Neurophysiol.

[32] Girardin CC, Martin KA (2009). Cooling in cat visual cortex: stability of orientation selectivity despite changes in responsiveness and spike width.. Neuroscience.

[33] de Polavieja GG, Harsch A, Kleppe I, Robinson HP, Juusola M (2005). Stimulus history reliably shapes action potential waveforms of cortical neurons.. J Neurosci.

[34] Kress GJ, Mennerick S (2009). Action potential initiation and propagation: upstream influences on neurotransmission.. Neuroscience.

[35] Kiyatkin EA (2010). Brain temperature homeostasis: physiological fluctuations and pathological shifts.. Front Biosci.

[36] Logothetis NK, Kayser C, Oeltermann A (2007). In vivo measurement of cortical impedance spectrum in monkeys: implications for signal propagation.. Neuron.

[37] Terzuolo CA, Araki T (1961). An analysis of intra- versus extracellular potential changes associated with activity of single spinal motoneurons.. Ann N Y Acad Sci.

[38] Gentet LJ, Williams SR (2007). Dopamine gates action potential backpropagation in midbrain dopaminergic neurons.. J Neurosci.

[39] Karlsen AS, Pakkenberg B (2011). Total numbers of neurons and glial cells in cortex and basal ganglia of aged brains with Down syndrome–a stereological study.. Cereb Cortex.

