# Supplementary figures and images for: Action Potential Waveform Variability Limits Multi-Unit Separation in Freely Behaving Rats
Source: PLoS One. 2012 Jun 12;7(6):e38482. doi: 10.1371/journal.pone.0038482 (PMC3373584; doi:10.1371/journal.pone.0038482)

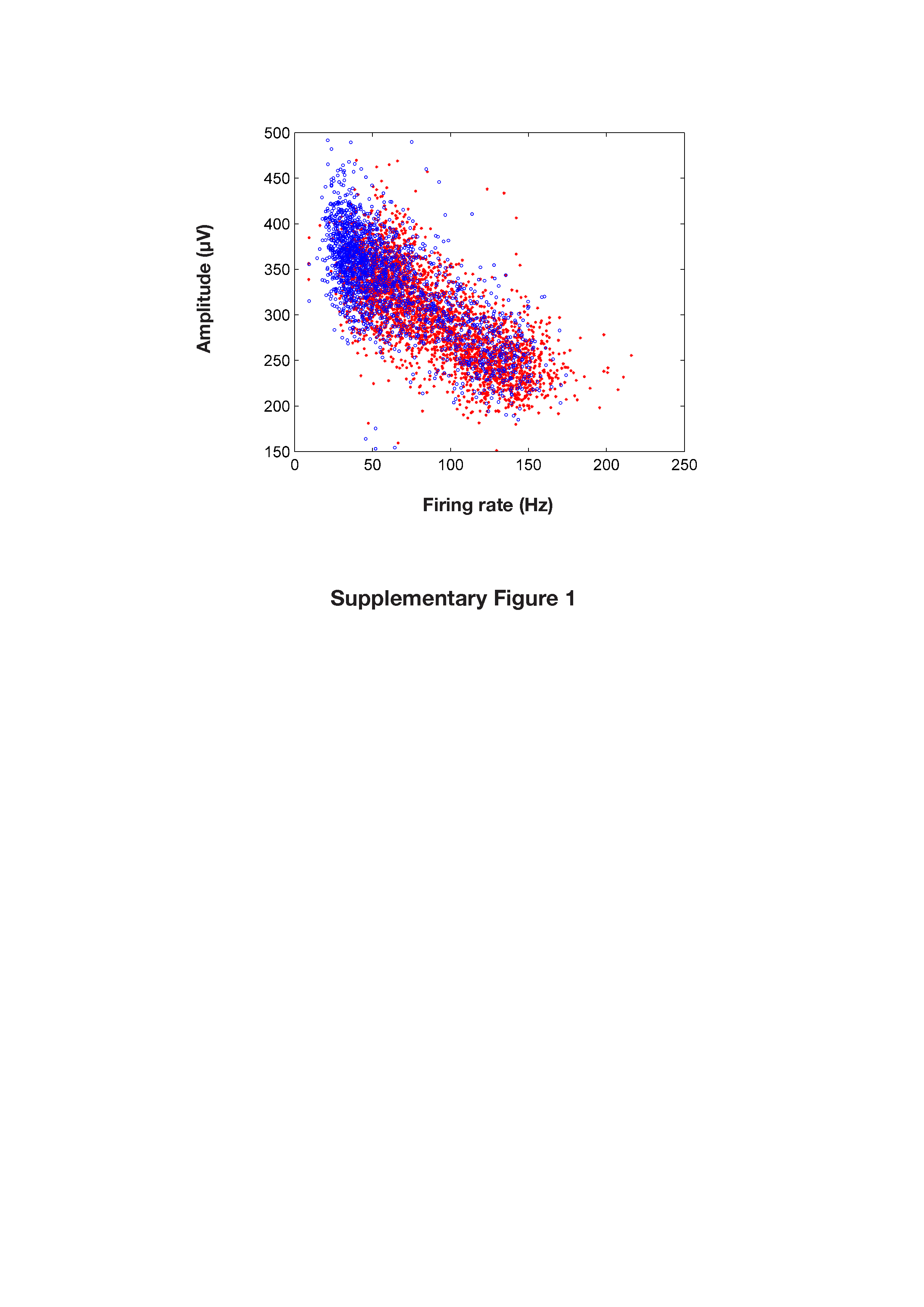

Supplement: Figure S1 — Action potential waveform amplitude to firing frequency variation for one neuron, recorded in unrestrained non-anaesthetised conditions. Blue circles and red dots represent action potentials recorded during baseline activity or glutamate iontophoresis respectively. Notice that glutamate iontophoresis reduces the likelihood of activity at low firing frequencies but does not alter the waveform amplitude or cause significant activity at firing frequencies higher than baseline. (TIFF) [file pone.0038482.s001.tiff]

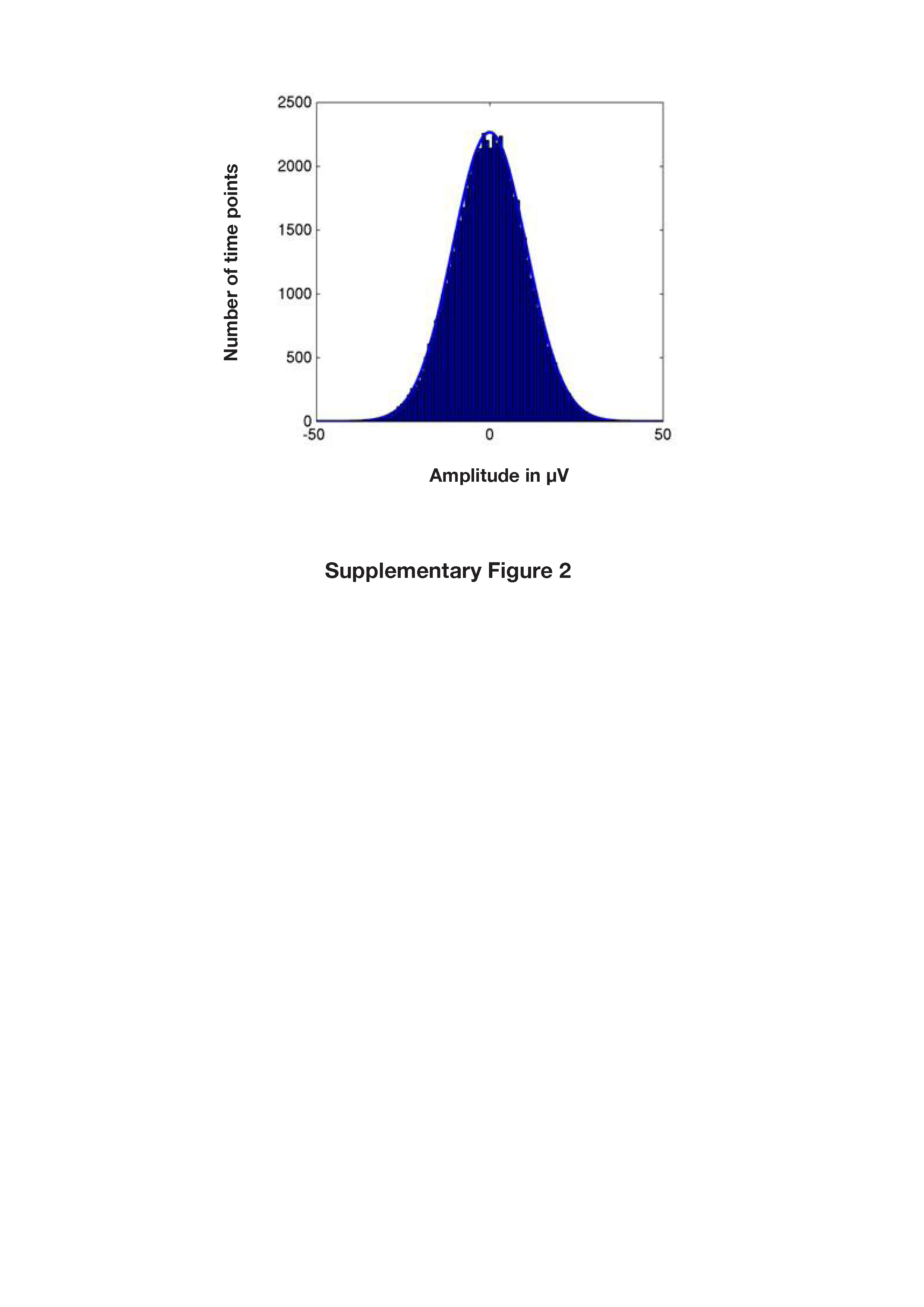

Supplement: Figure S2 — Noise distribution for the first 2 seconds of recording in the substantia nigra pars reticulata in an awake unrestrained rat with Gaussian fitted. A Kolmogorov-Smirnov test supports a Gaussian noise distribution (p = 0.5418). (TIFF) [file pone.0038482.s002.tiff]

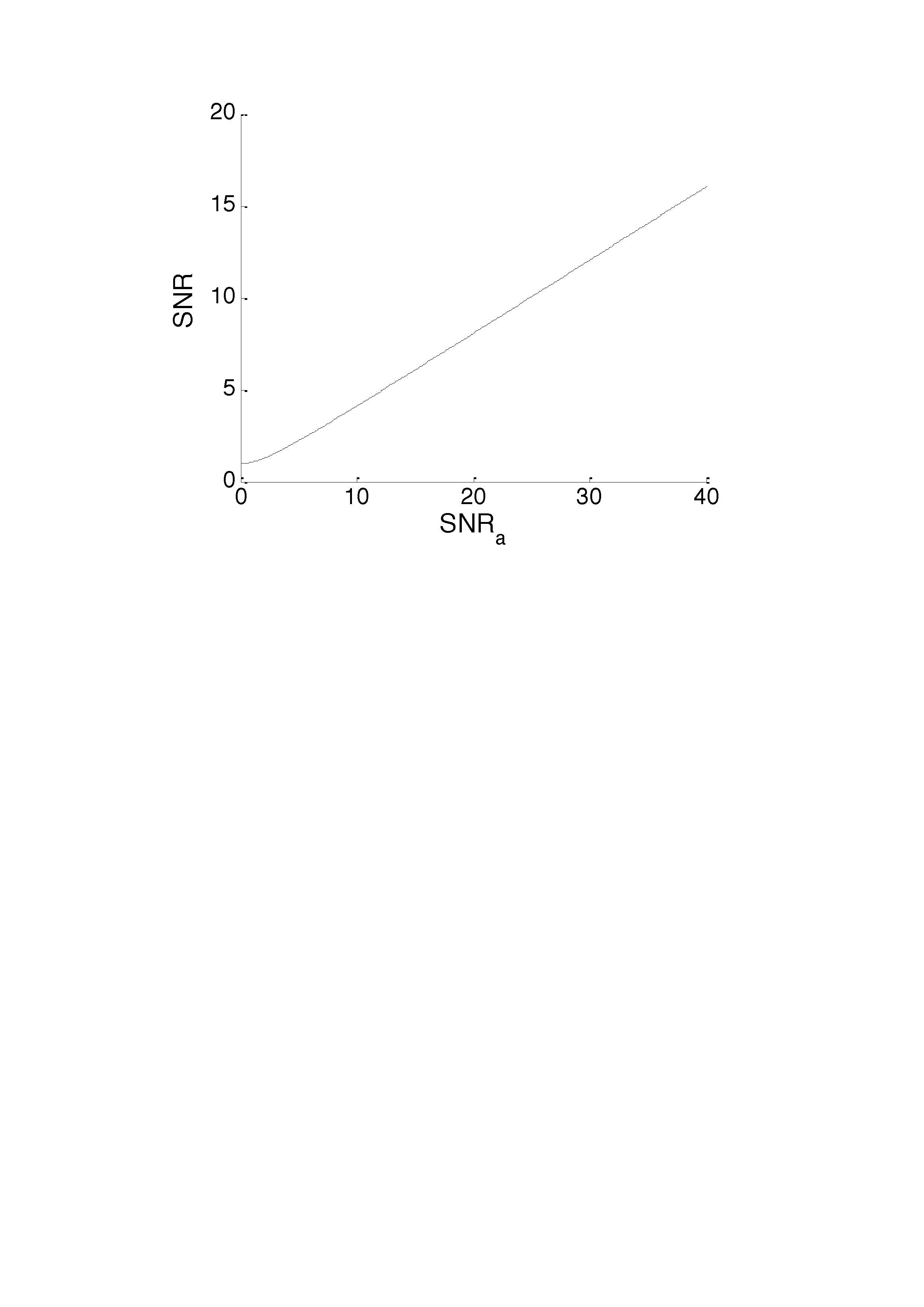

Supplement: Figure S3 — Graphic representation of SNR and SNRa relation; see text for details. (TIFF) [file pone.0038482.s003.tiff]

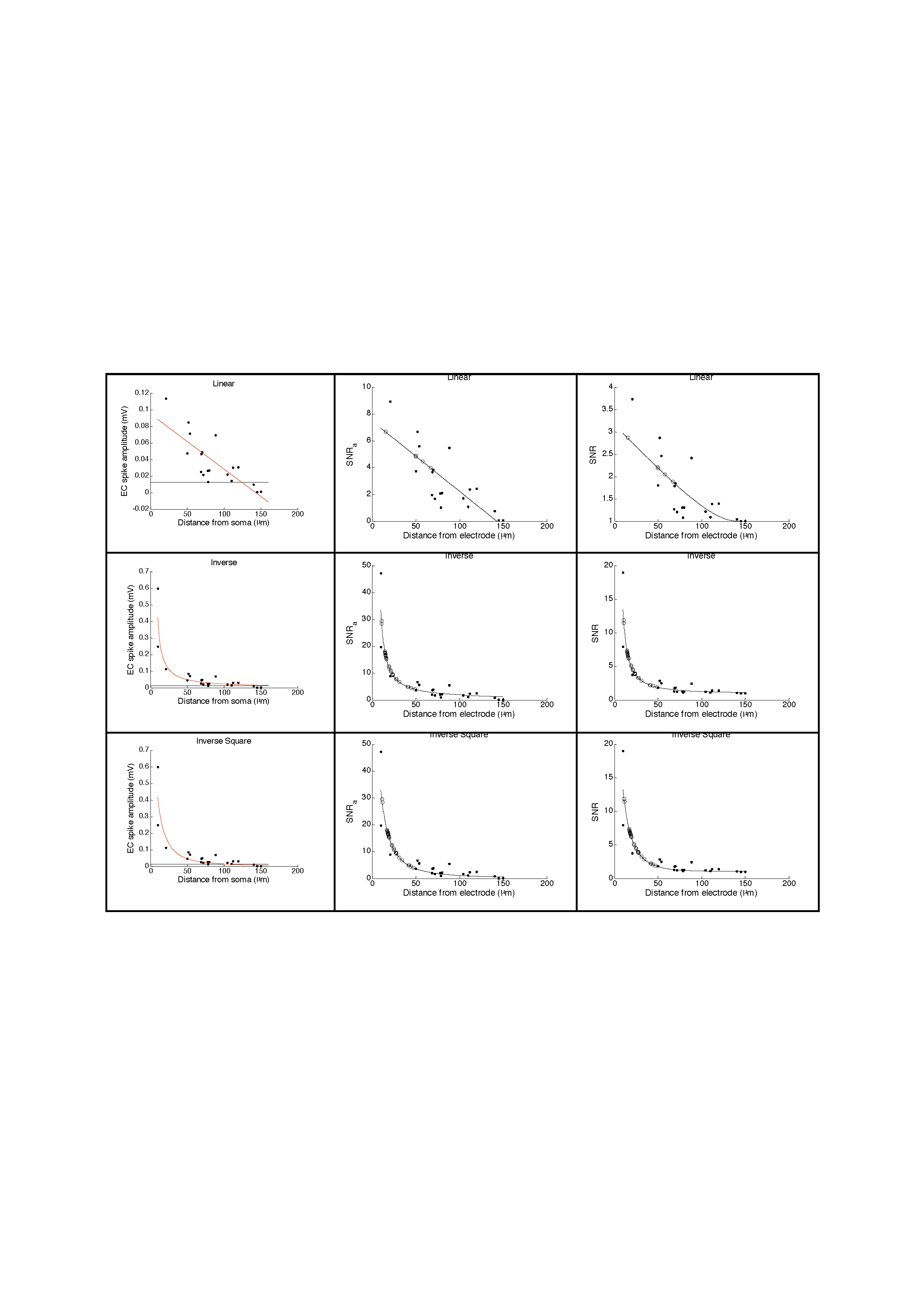

Supplement: Figure S4 — Model fits to extracellular spike amplitude data, and predicted and (S15). For consistency, all SNR curves start at 10 µm (assumed radial distance of cell at 600 µV). The extracellular spike amplitude (left column) refers to the average maximum deflection from baseline. The RMS noise (left column) is shown as dashed lines. The , and estimated distances are shown for data used in this work for the non-linear models (circles). The peak deflection of all mean spike shapes were between 50.4% and 68.6% of the peak-to-peak amplitude. Data are courtesy of the Buzsaki group from the Collaborative Research in Computational Neuroscience data-sharing website (crcns.org). (TIFF) [file pone.0038482.s004.tiff]

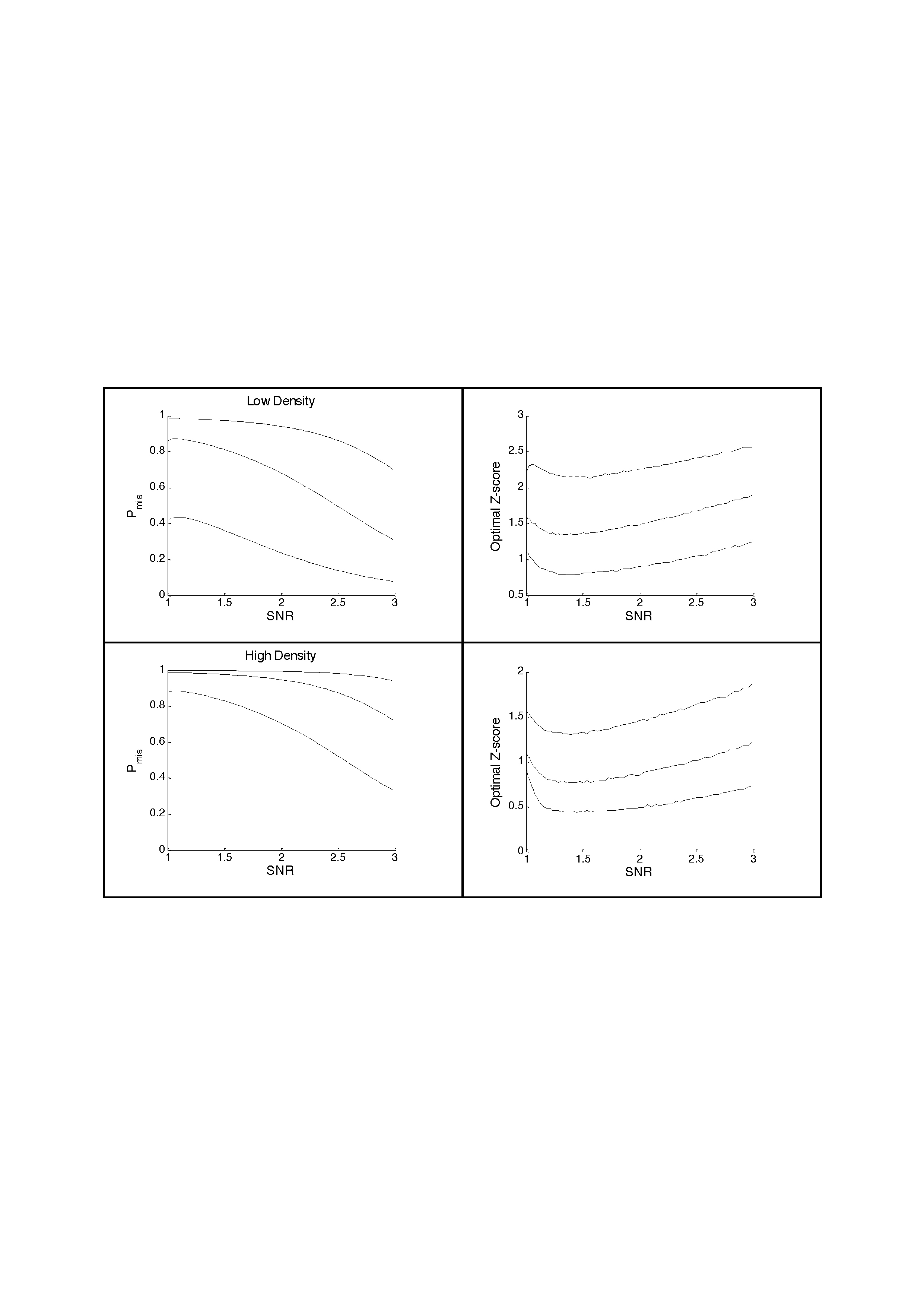

Supplement: Figure S5 — For the linear model, expected probability of misclassification, Pmis , is unacceptably high for almost any attainable recording SNR. Left: Pmis for low density (top) and high density (bottom) brain regions as a function of recording SNR. Top trace: 100% of neurons firing; middle trace: 10% firing; bottom trace: 1% firing. Right: Optimal cluster Z-score sizes for each case (note that the top-to-bottom order is reversed; that is, the traces represent 1%, 10% then 100% of neurons firing). At low firing rate and low density, the lower average misclassification rate is partly due to the linear drop in spike amplitude, which means that there are relatively few cells with low SNR. On average there is only approximately one cell firing (4 cells per 50 µm radius hemisphere = 108 cells per 150 µm hemisphere, which at 1% firing is about 1 cell). For 90% success rate, an SNR of 2.8 is sufficient in this case. Notice, however, that the highest attainable SNR for the linear model is about 3, which does not fit with experimental observations. (TIFF) [file pone.0038482.s005.tiff]

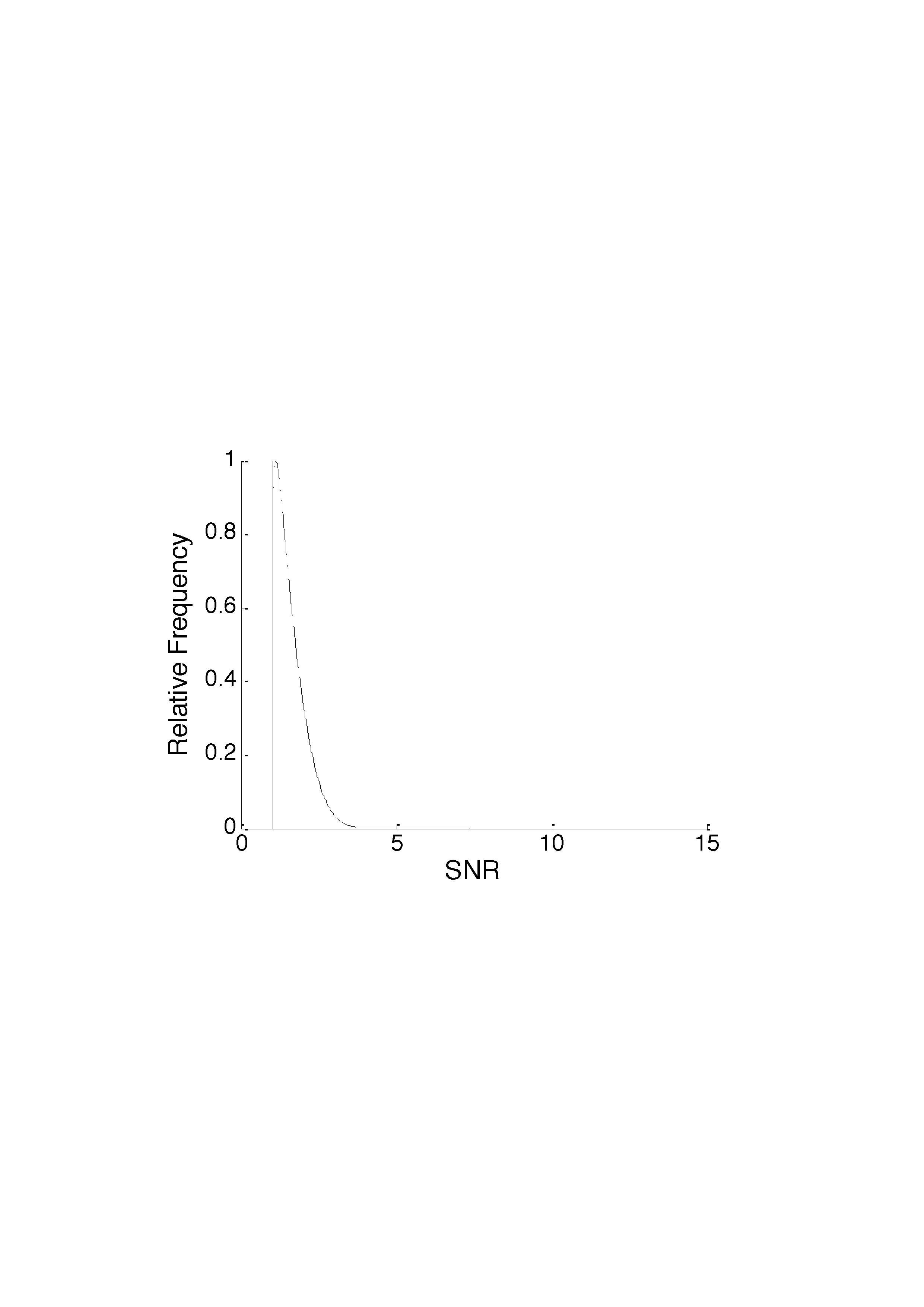

Supplement: Figure S6 — Expected relative frequency of the SNR of neural recordings for randomly placed electrodes, for the linear model. (TIFF) [file pone.0038482.s006.tiff]

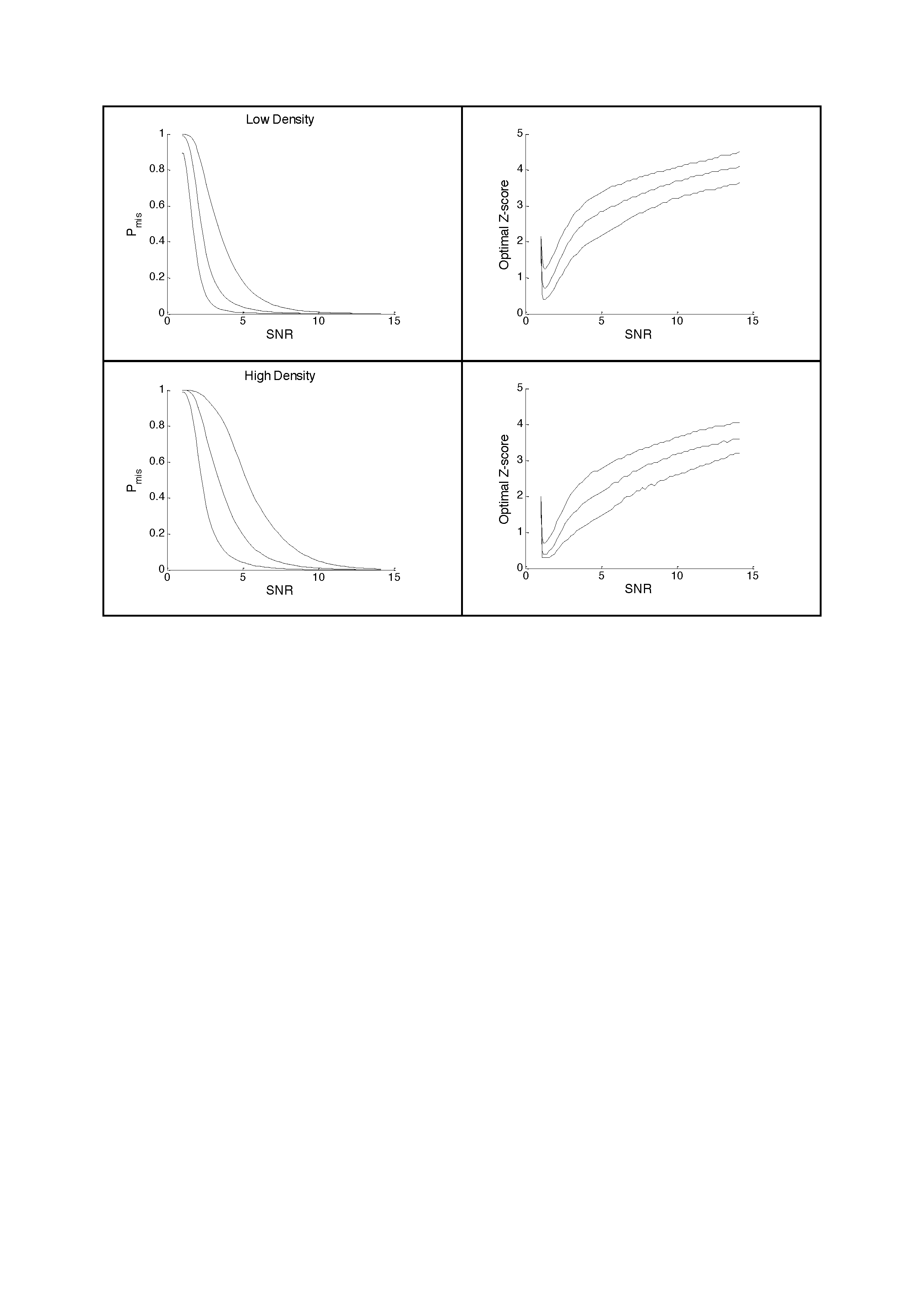

Supplement: Figure S7 — For the inverse model, expected probability of misclassification, Pmis , approaches 1 at low recording SNRs. Left: Pmis for low density (top) and high density (bottom) brain regions as a function of recording SNR. Top trace: 100% of neurons firing; middle trace: 10% firing; bottom trace: 1% firing. Right: Optimal cluster Z-score sizes for each case (note that the top-to-bottom order is reversed; that is, the traces represent 1%, 10% then 100% of neurons firing). (TIFF) [file pone.0038482.s007.tiff]

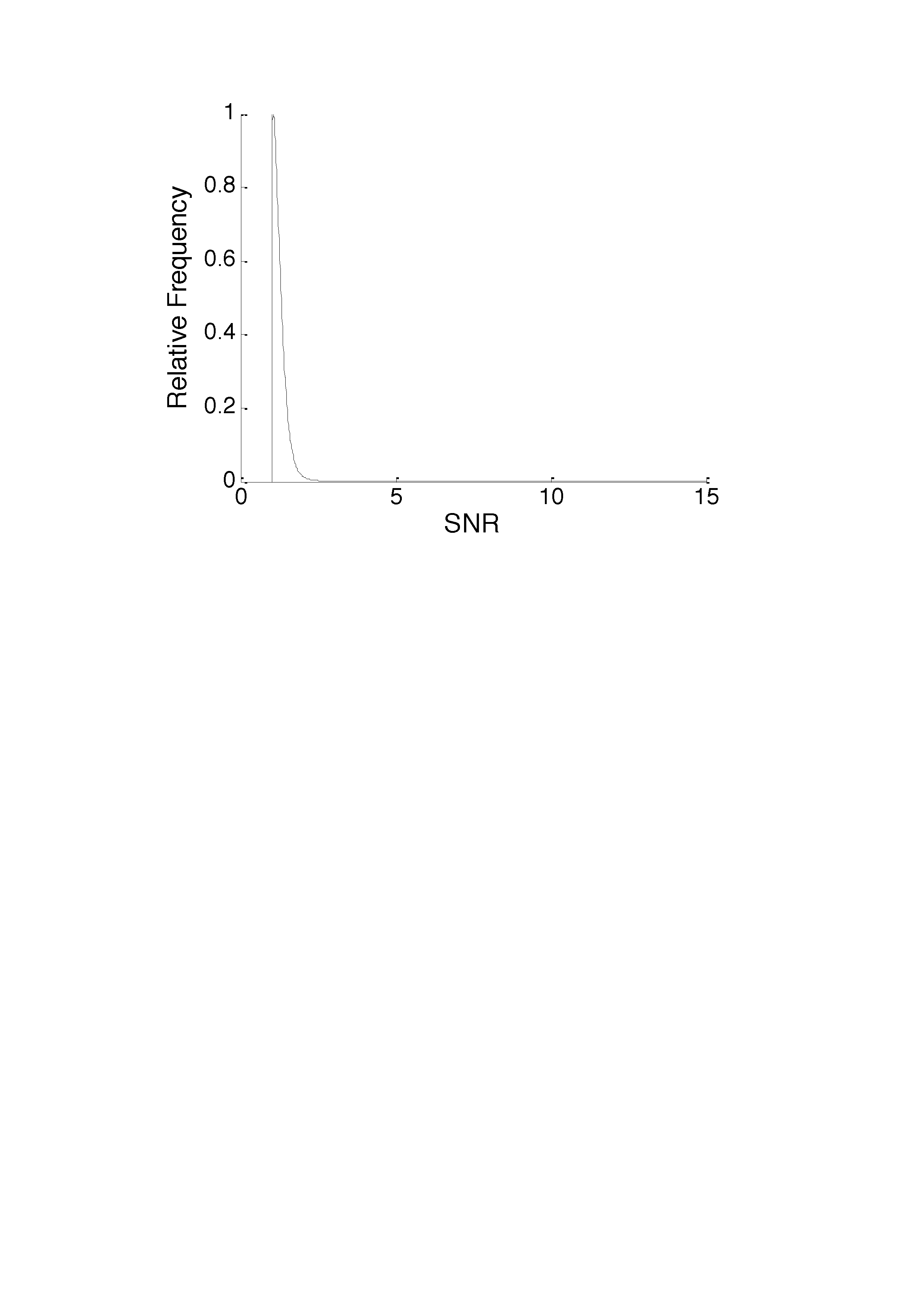

Supplement: Figure S8 — Expected relative frequency of the SNR of neural recordings for randomly placed electrodes, for the inverse model. (TIFF) [file pone.0038482.s008.tiff]

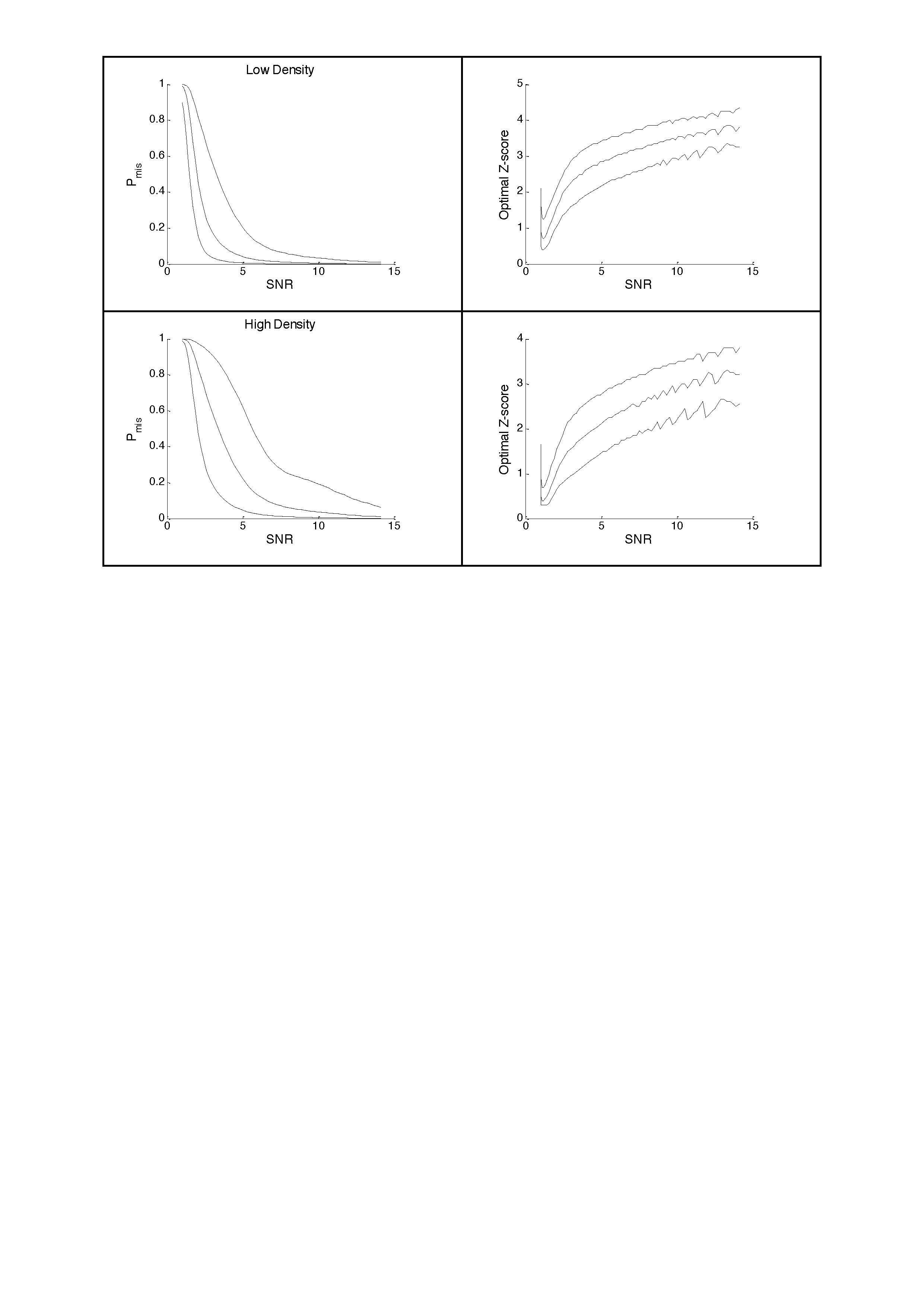

Supplement: Figure S9 — For the inverse square model, expected probability of misclassification, Pmis , approaches 1 at low recording SNRs. Left: Pmis for low density (top) and high density (bottom) brain regions as a function of recording SNR. Top trace: 100% of neurons firing; middle trace: 10% firing; bottom trace: 1% firing. Right: Optimal cluster Z-score sizes for each case (note that the top-to-bottom order is reversed; that is, the traces represent 1%, 10% then 100% of neurons firing). (TIFF) [file pone.0038482.s009.tiff]

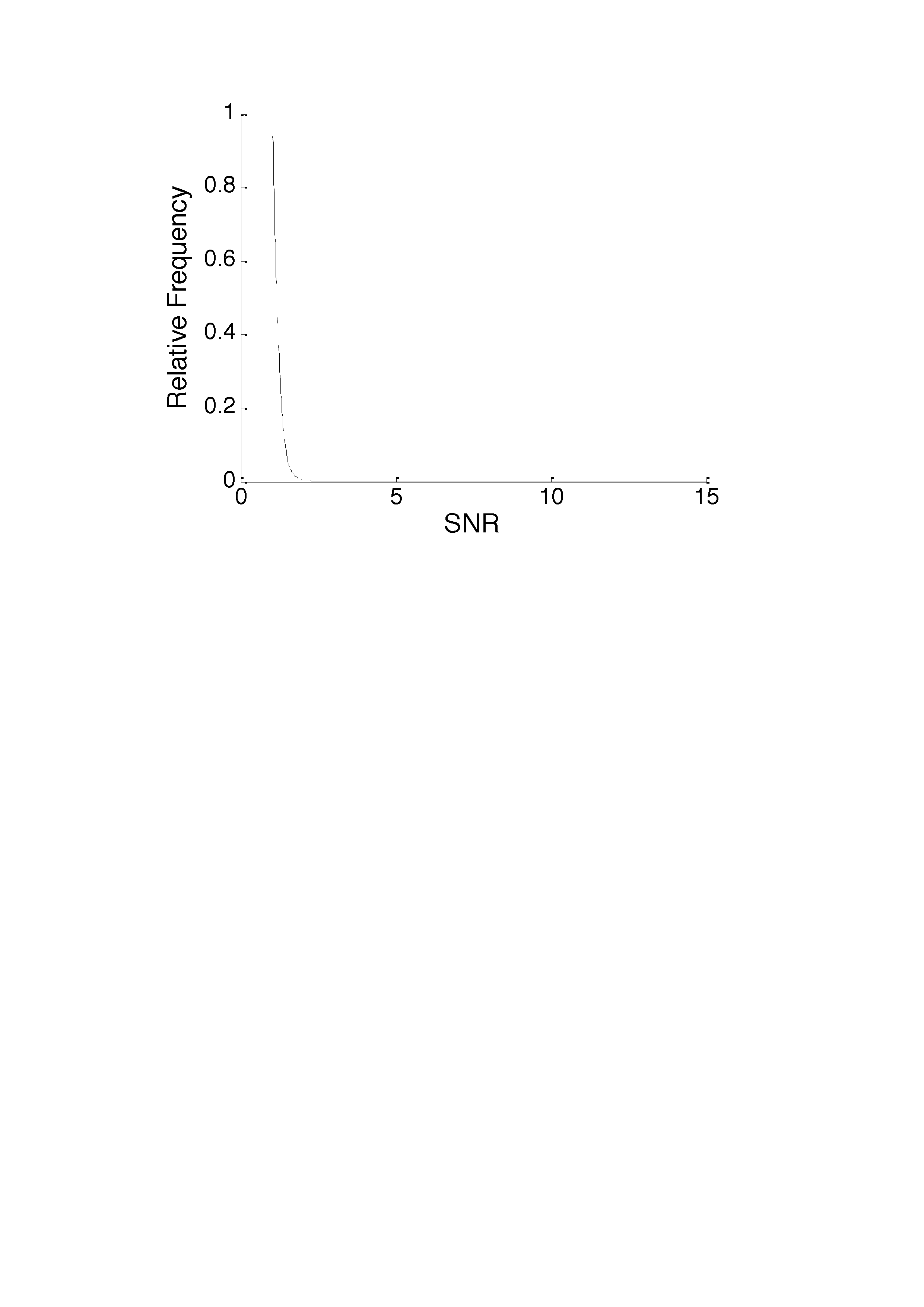

Supplement: Figure S10 — Expected relative frequency of the SNR of neural recordings for randomly placed electrodes, for the inverse square model. (TIFF) [file pone.0038482.s010.tiff]

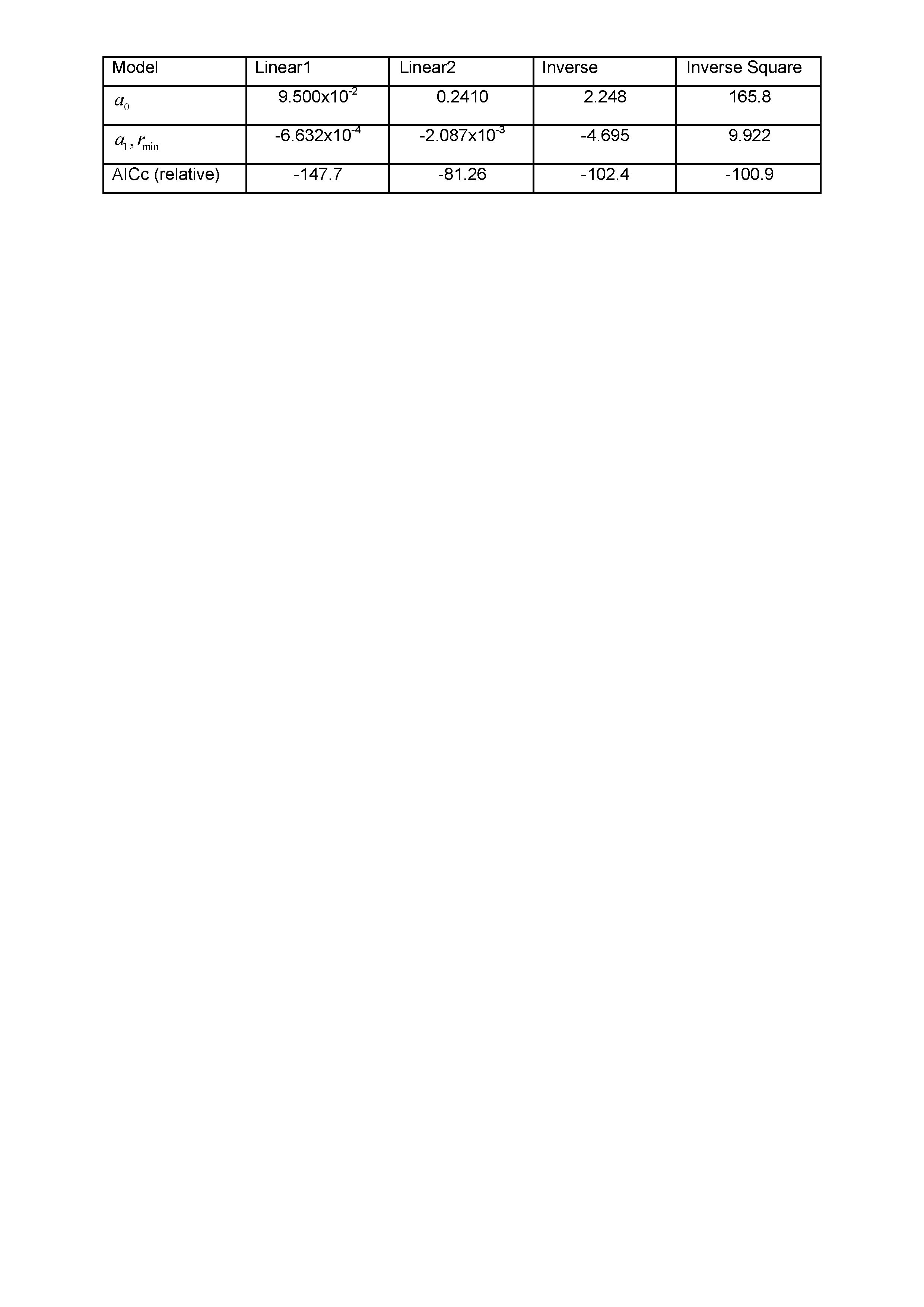

Supplement: Table S1 — Fit parameters and the corrected Akaike Information Criterion for each model. (TIFF) [file pone.0038482.s015.tiff]

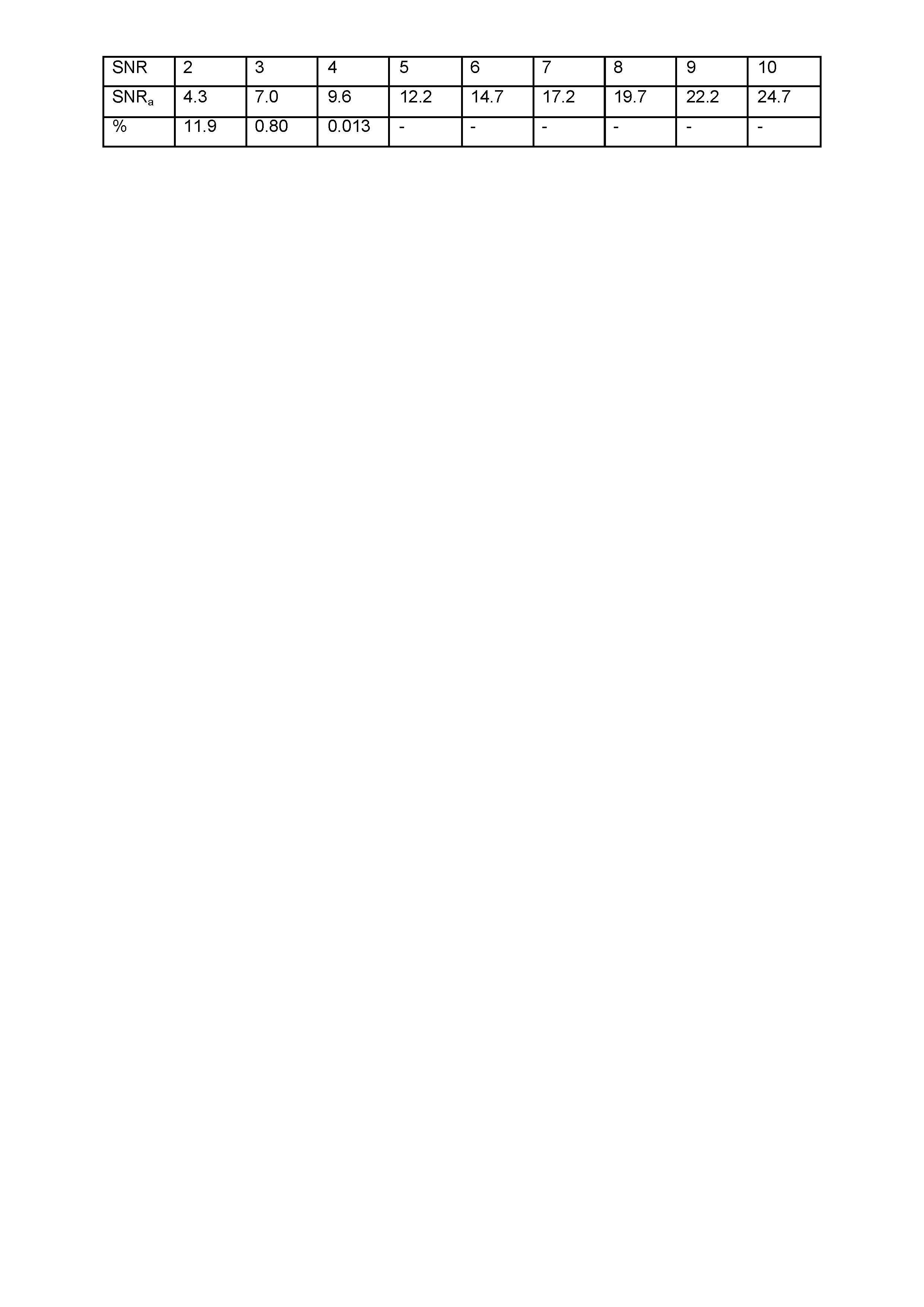

Supplement: Table S2 — Percentage of cells above a given SNR assuming random cell and electrode positions, for the linear model. (TIFF) [file pone.0038482.s016.tiff]

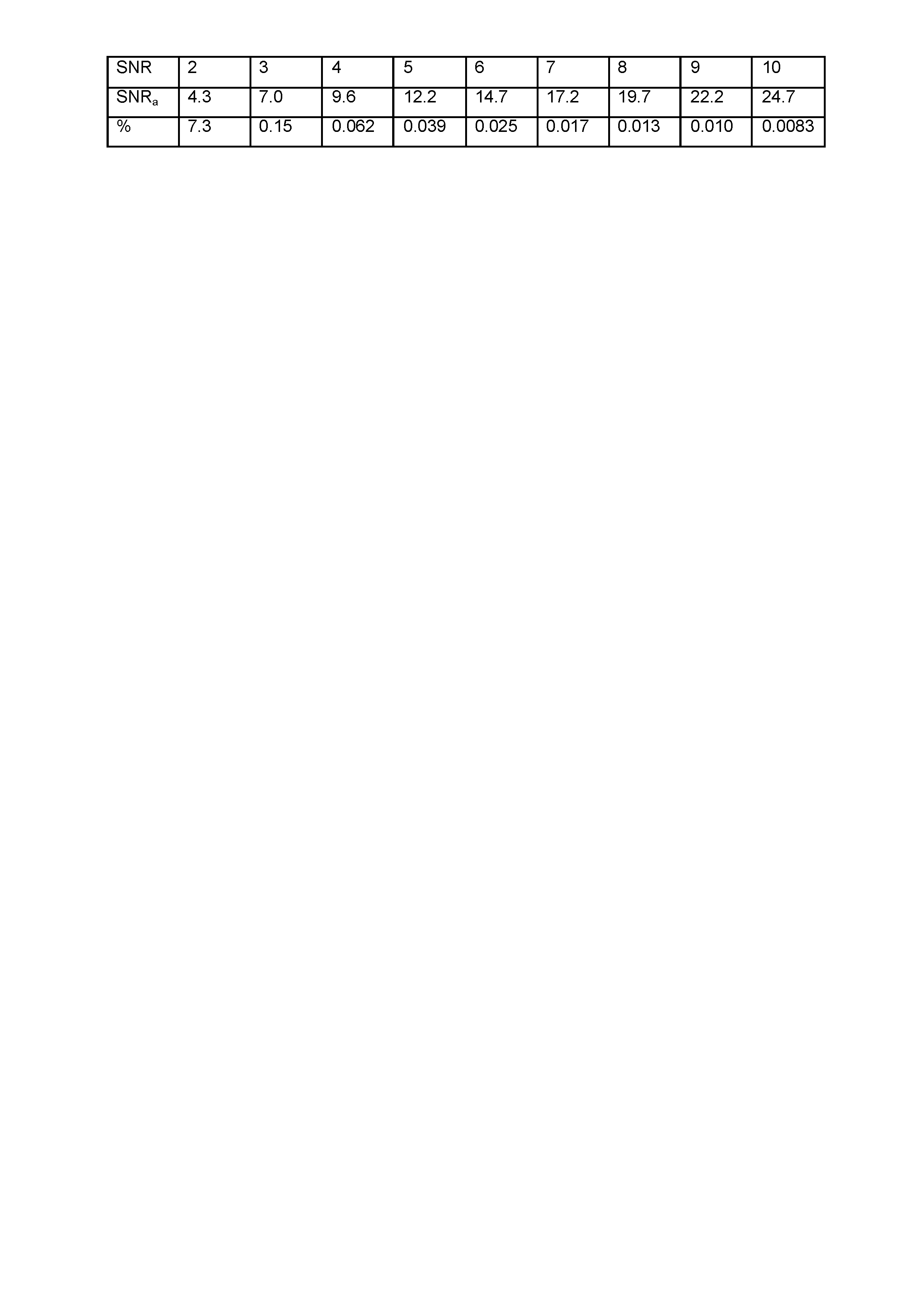

Supplement: Table S3 — Percentage of cells above a given SNR assuming random cell and electrode positions, for the inverse model. (TIFF) [file pone.0038482.s017.tiff]

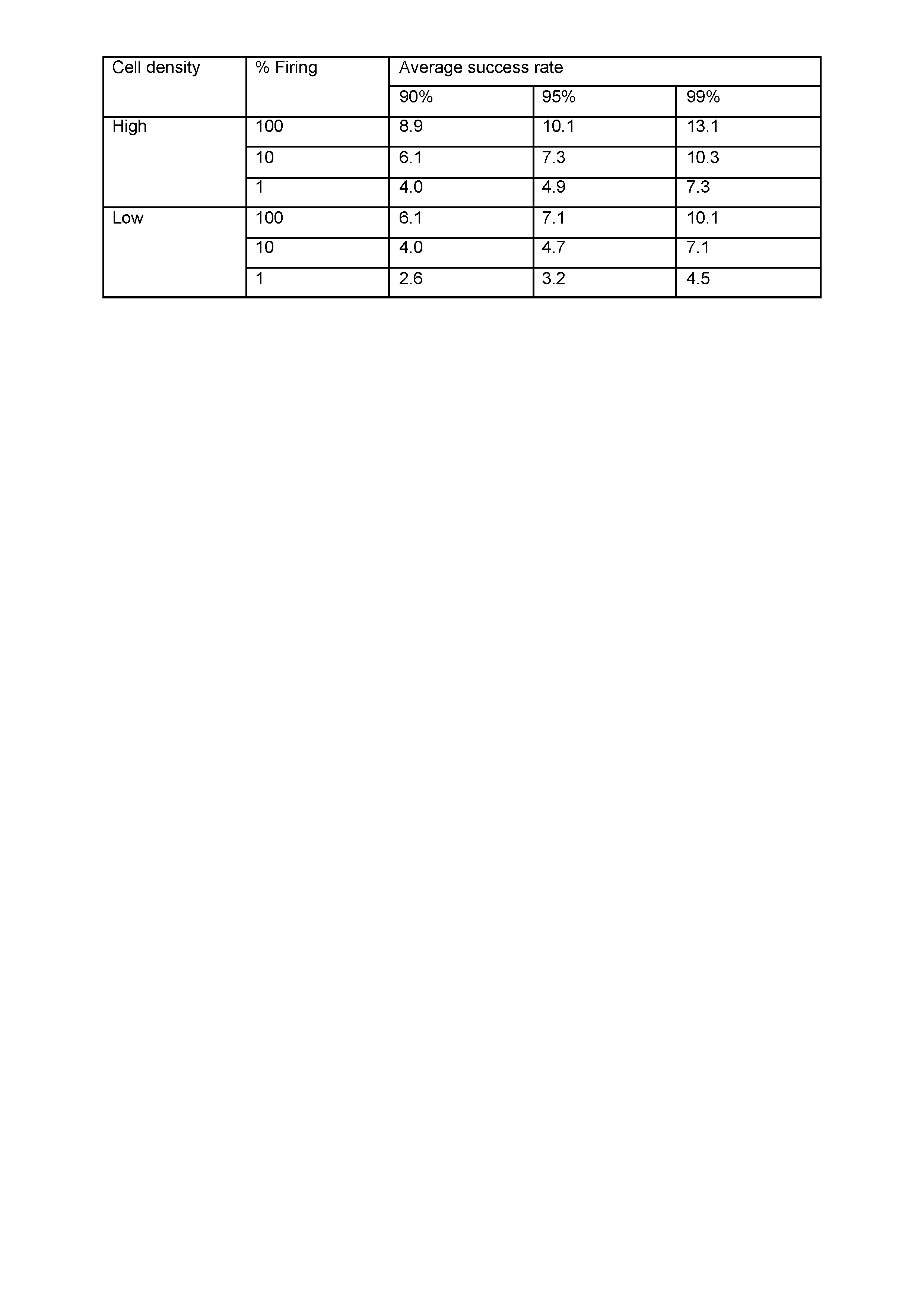

Supplement: Table S4 — Minimum SNR required to achieve a given success rate defined as , for the inverse model. (TIFF) [file pone.0038482.s018.tiff]

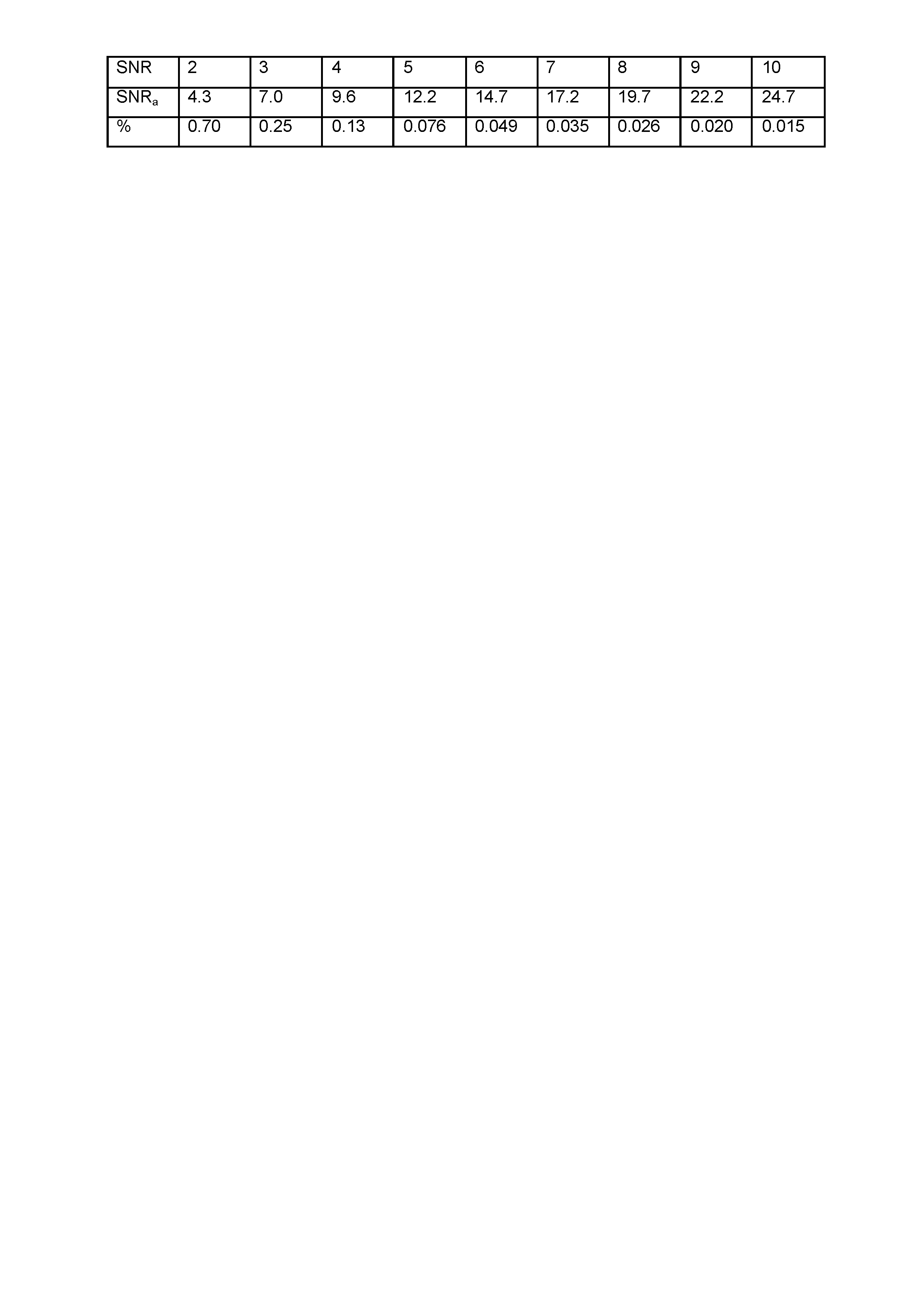

Supplement: Table S5 — Percentage of cells above a given SNR assuming random cell and electrode positions, for the inverse square model. (TIFF) [file pone.0038482.s019.tiff]

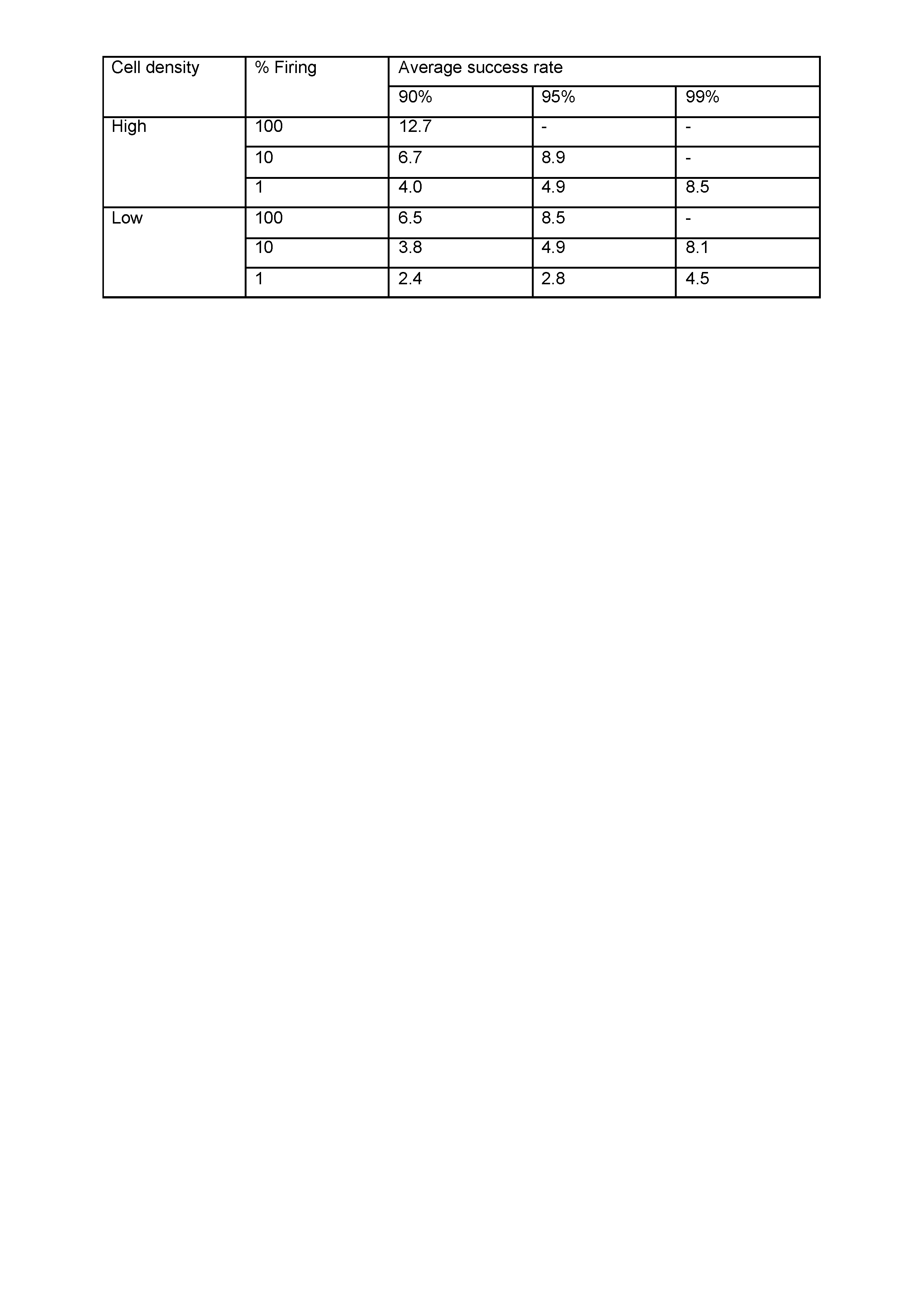

Supplement: Table S6 — Minimum SNR required to achieve a given success rate defined as , for the inverse square model. (TIFF) [file pone.0038482.s020.tiff]
