# Supplementary material for: Action Potential Waveform Variability Limits Multi-Unit Separation in Freely Behaving Rats
Source: PLoS One. 2012 Jun 12;7(6):e38482. doi: 10.1371/journal.pone.0038482 (PMC3373584; doi:10.1371/journal.pone.0038482)
Supplement: Material S1 — Relating spike-shape variability to SNR. (DOC) [file pone.0038482.s011.doc]

**Supplementary material 1** **– Relating spike-shape variability to SNR**

Following the definition of spike similarity/variability used in this work, we consider spike vectors normalised such that their RMS amplitudes are always unity. Suppose the average waveform is $y_{0}=a_{0}f\left(t\right)$. The mean normalised amplitude $RMS\left(y_{0}\right)=a_{0}k=1$, where $k$ is a constant which depends on the shape of the spike. Hence $a_{0}=1/k$.

Now, an individual spike has noise $y_{1}=a_{1}f\left(t\right)+\varepsilon$ where $\varepsilon$ is a zero-mean random variable with variance $\sigma_{\varepsilon}^{2}$ (*i.e.*, noise – which may or may not be Gaussian). As the spike vector is normalised, $RMS\left(y_{1}\right)=\sqrt{k^{2}a_{1}^{2}+\sigma_{\varepsilon}^{2}}=1$ and $SNR=1/\sigma_{\varepsilon}$ (RMS form). Rearranging we get $a_{1}^{2}=\left(1-\sigma_{\varepsilon}^{2}\right)/k^{2}$ and so $a_{1}=\sqrt{1-1/SNR^{2}}/k=a_{0}\sqrt{1-1/SNR^{2}}$. Therefore

$$\begin{matrix} \left\langle variability\right\rangle=1-\left\langle y_{0}\cdot y_{1}\right\rangle\\ =1-\left\langle\left[a_{0}f\left(t\right)\right]\cdot\left[a_{0}\sqrt{1-\frac{1}{SNR^{2}}}f\left(t\right)+\varepsilon\right]\right\rangle\\ =1-\sqrt{1-\frac{1}{SNR^{2}}}\left\langle\left[a_{0}f\left(t\right)\right]\cdot\left[a_{0}f\left(t\right)\right]\right\rangle\\ =1-\sqrt{1-\frac{1}{SNR^{2}}} \end{matrix}$$

This ideal relationship is independent of spike waveform, but does assume that the waveform (shape and amplitude) does not vary during a recording. Small variations in amplitude lead to an inflation of the estimate of the SNR. This is because the RMS function weights higher amplitudes more heavily than lower amplitudes, whereas the dot product is unbiased with respect to amplitude. The result is that the relationship between variability and SNR is shifted to the right (consistent with simulation results, see below).

However, the relative contribution of noise to the shape vector is critically dependent on the SNR, which is a more important determinant of normalised shape variability than variations in amplitude. Therefore, the distribution in normalised shape space is determined largely by the noise, which is assumed to be an independent but differently distributed (IDD) Gaussian. Hence (S1) holds even though we do not know *a priori* how the spike amplitude varies within a recording, or how the average spike shape varies between neurons. The remaining unexplained variability is therefore unlikely to be due to noise or simple amplitude variations accounted for by a scale factor. The most likely explanation is intrinsic variability in shape, which could be due to changes in ion channel kinetics. The latter is supported by the observation of changes that in spike kinetic parameters with changing spiking frequency.
