# Supplementary material for: Action Potential Waveform Variability Limits Multi-Unit Separation in Freely Behaving Rats
Source: PLoS One. 2012 Jun 12;7(6):e38482. doi: 10.1371/journal.pone.0038482 (PMC3373584; doi:10.1371/journal.pone.0038482)
Supplement: Material S2 — Modelling SNR in terms of spike amplitude variation and spike shape. (DOC) [file pone.0038482.s012.doc]

**Supplementary material 2 – Modelling SNR in terms of spike amplitude variation and spike shape**

*Definitions*

1) $a$ denotes the true amplitude which is the maximum deflection of a spike from baseline.

2) $a^{*}$ denotes the measured amplitude.

2) $f$ denotes the true waveform (over the time window defined as being part of the spike).

3) $f^{*}$ denotes the measured waveform.

3) denotes the expectation over the distribution of $a$ (over the entire recording).

4) $\varepsilon$ denotes noise which is independent of spike amplitude or shape.

5) Without loss of generality, assume that $\left\langle\varepsilon\right\rangle=0$ and $\left\langle\varepsilon^{2}\right\rangle=\sigma_{\varepsilon}^{2}$. NB: if $\left\langle\varepsilon\right\rangle\neq0$, it is equivalent to a constant baseline shift, which is removed in the analysis.

*SNR model*

At time *t* during a spike, it is assumed that

$$f^{*}\left(t\right)=f\left(t\right)+\varepsilon\left(t\right)$$

By definition, the SNR of each recording is

$$SNR=\sqrt{\frac{\left\langle\left(f^{*}\right)^{2}\right\rangle_{a}}{\sigma_{\varepsilon}^{2}}}\approx\sqrt{\frac{\sum_{i} \overline{\left(f_{i}^{*}\right)^{2}}}{n\sigma_{\varepsilon}^{2}}}$$

where *n* is the total number of spikes. For simplicity, the true noise variance is assumed to be known due to the large number of baseline noise points available.

Furthermore,

$$a_{i}^{*}=\max\left(\left|f_{i}^{*}\right|\right)$$

where the subscript *i* denotes the spike number in a recording. Ideally, the maximum deflection is measured at the time of the true maximum.

From the definitions above, the true mean squared amplitude of a cell’s spikes is

$$\left\langle a^{2}\right\rangle=\left\langle\max\left(\left|f\right|\right)^{2}\right\rangle_{a}=\left\langle\max\left(f^{2}\right)\right\rangle_{a}=\mu_{a}^{2}+\sigma_{a}^{2}$$

where $\mu_{a}=\left\langle\max\left(\left|f\right|\right)\right\rangle_{a}$ , $\sigma_{a}^{2}$ is the variance of the true amplitude. In practice, the mean amplitude can also be calculated as $\mu_{a}=\max\left(\left|f_{0}\right|\right)$ where $f_{0}=\left\langle f\right\rangle_{a}$ is the average waveform of a recording. For large spike number *n*, $f_{0}$ is essentially noise-free.

However, the measured mean squared amplitude in a recording is affected by noise:

This assumes that the true peak of each spike is measured. It is worth noting that, in general, $\left\langle a^{*2}\right\rangle>\left\langle a^{2}\right\rangle>\mu_{a}^{2}$.

For a constant spike shape, $\sqrt{\left\langle f_{0}^{2}\right\rangle}/\mu_{a}=k$ depends on the intrinsic shape of the spike. Spikes which are more peaked will tend to have smaller values of *k*, whereas broader spikes tend to have larger values of *k*. For each spike, $\left\langle f_{i}^{2}\right\rangle=\left(ka_{i}\right)^{2}$ thus

$$\left\langle f^{2}\right\rangle_{a}=\left\langle\left(ka\right)^{2}\right\rangle_{a}=k^{2}\left(\mu_{a}^{2}+\sigma_{a}^{2}\right)$$

For a sine wave, *i.e.*, $f\propto\sin\left(t\right)$, $k=1/\sqrt{2}$; for a zero-mean square wave, $k=1$. NB: in practice, this shape parameter can be estimated in two simple ways, *e.g.*, or $\widehat{k}=\sqrt{\left\langle f_{mean\_spike}^{2}\right\rangle}/\mu_{mean\_spike}$. From our recordings, $\overline{k}\approx0.40$(see Figure S3 for the relationship between *SNR* and *SNR_a_*). For convenience, this shape parameter can be written simply as *k*.

For all measured spikes,

$$\left\langle\left(f^{*}\right)^{2}\right\rangle_{a}=\left\langle\left(f+\varepsilon\right)^{2}\right\rangle_{a}=\left\langle f^{2}+2f\varepsilon+\varepsilon^{2}\right\rangle_{a}=\left\langle f^{2}\right\rangle_{a}+\sigma_{\varepsilon}^{2}$$

Thus,

$$SNR^{2}=\frac{\left\langle\left(f^{*}\right)^{2}\right\rangle_{a}}{\sigma_{\varepsilon}^{2}}=\frac{k^{2}\left(\mu_{a}^{2}+\sigma_{a}^{2}\right)}{\sigma_{\varepsilon}^{2}}+1$$

For each neuron, it is reasonable to assume that the baseline can be estimated relatively precisely so that noise only contributes to the estimate of the peak deflection. Hence the measured ratio is

$$\nu^{2}=\frac{\sigma_{a}^{*2}}{\mu_{a}^{2}}=\frac{\sigma_{a}^{2}+\sigma_{\varepsilon}^{2}}{\mu_{a}^{2}}$$

This depends on the relative level of the noise, and is therefore not constant. However, assuming

$$\gamma=\frac{\sigma_{a}}{\mu_{a}}$$

is constant, (S11)→(S9) gives

$$SNR=\sqrt{\frac{k^{2}\mu_{a}^{2}\left(1+\gamma^{2}\right)}{\sigma_{\varepsilon}^{2}}+1}$$

Combining with (S10) gives

which is a measure of the intrinsic amplitude variability of each cell ($\mu_{\gamma}\pm SE_{\gamma}\approx0.14\pm0.01$).

For convenience, we include a second definition of SNR which is the ratio of the average maximum deflection to the RMS noise *i.e.*,

$$SNR_{a}=\frac{\mu_{a}}{\sigma_{\varepsilon}}\approx\frac{\sum_{i} a^{*}_{i}}{n\sigma_{\varepsilon}}$$

where *n* is the total number of spikes. This definition allows the use of peak absolute amplitude (*e.g.* Henze *et al.* 2000 + personal communication) and does not require defining the precise start and end of the signal in question, a possible advantage in spike analysis.

Combining (S12) and (S14), the standard *SNR* is related to *SNR_a_* by

$$SNR=\sqrt{k^{2}\left(1+\gamma^{2}\right)SNR_{a}^{2}+1}$$
