# Supplementary material for: Action Potential Waveform Variability Limits Multi-Unit Separation in Freely Behaving Rats
Source: PLoS One. 2012 Jun 12;7(6):e38482. doi: 10.1371/journal.pone.0038482 (PMC3373584; doi:10.1371/journal.pone.0038482)
Supplement: Material S3 — Relationship between SNR and distance from electrode tip. (DOC) [file pone.0038482.s013.doc]

**Supplementary material 3 - Relationship between SNR and distance from electrode tip**

From Fig 6A of Henze *et al.* (2000), data pairs of extracellular spike amplitude and distance from the soma were extracted. According to the authors (personal communication) these were maximum deflections *i.e.*, $\mu_{a}$. In the main text, the authors also reported extracellular amplitudes >250μV and as high as 600μV. Due to difficulties with achieving concomitant labelling, presumably due to the close proximity of the extracellular electrode to the soma, the distance measurements from the soma were not available. However, the presence of such large extracellular amplitudes clearly demonstrates nonlinearity in the relationship between extracellular amplitude and distance. For function-fitting, we included these extracellular amplitudes in the two non-linear models, and assigned a distance approximately midway to the minimum reported distance of just over 20μm (*i.e.* 10μm). The baseline noise $\sigma_{\varepsilon}$ was estimated from the dotted line in Fig 6A of Henze *et al.* (2000). Four models relating $\mu_{a}$ to *r* were investigated.

Linear1 (excludes 250 and 600 μV points) and Linear2

$$\mu_{a}=a_{0}-a_{1}r$$

Inverse

$$\mu_{a}=\frac{a_{0}}{r+r_{\min}}$$

Inverse Square

$$\mu_{a}=\frac{a_{0}}{\left(r+r_{\min}\right)^{2}}$$

Using the corrected Akaike Information Criterion, the Inverse Square model is 0.48 times as likely as the Inverse model to minimise information loss (see Table S1). In other words, the Inverse model is a better fit than the Inverse Square model (which is far better than Linear2). However, the fitted $r_{\min}$ is about -5 μm (see Figure S4).

The relative likelihood is given by $l=Exp\left(\left(AIC_{\min}-AIC_{i}\right)/2\right)$. Due to the obviously poor fit and low explanatory value, the Linear2 model was excluded from further analysis.
