# Supplementary material for: Action Potential Waveform Variability Limits Multi-Unit Separation in Freely Behaving Rats
Source: PLoS One. 2012 Jun 12;7(6):e38482. doi: 10.1371/journal.pone.0038482 (PMC3373584; doi:10.1371/journal.pone.0038482)
Supplement: Material S4 — Unified probability of spike misclassification. (DOC) [file pone.0038482.s014.doc]

**Supplementary material 4 – Unified probability of spike misclassification**

Here we consider the entire volume of neural tissue within which spikes may be recorded (currently assumed to be within a hemisphere of radius 300 μm or when amplitude drops to 0, whichever occurs first). For a given cell density, we randomly distribute the cells within the hemisphere. Each cell’s radial distance, and hence SNR as well as maximum deflection amplitude, can be calculated as shown earlier.

In addition, the spike shape variability is considered as an independent variable. Applying the same arguments as before, we consider only the effect of noise on the shape (ignoring intrinsic shape variations which would in practice make classification more difficult). We know the lower bound for the average shape variability (due to noise only), and we make the additional assumption that noise is Gaussian. For any two neurons within the hemisphere of recordable tissue, their marginal shape distributions must also be Gaussian along the axis defined by their two mean spike shapes.

Now consider the joint amplitude–shape distribution of the spikes of one neuron *i.e.*, a neuron of interest. It is trivial to apply the model of amplitude (as a function of SNR) to describe the amplitude frequency distribution due to all other neurons in the recordable volume of tissue. In fact, based on the earlier assumptions, it is also true that the shape frequency distribution of all neurons *with respect to* the neuron of interest can also be modelled as an independent variable (along an orthogonal axis). Furthermore, the numbers of contaminating spikes (false positives) attributable to each neuron are independent of each other, and hence are additive. Finally, due to the assumption of sphericity in shape space, the number of excluded spikes (false negatives) is independent of the direction in shape space.

Taken together, this means that the frequency distribution of spikes from each neuron can be modelled as a bivariate Gaussian with a covariance matrix, the off-diagonal terms of which are zero. Furthermore, the combined frequency distribution in amplitude shape space can be represented by summing all bivariate Gaussians. Note that the overlap between the neuron of interest and all other neurons in shape space is meaningful, but this is not the case between each of the other neurons (see Figures S6, S8 and S10 and Tables S2, S3 and S5 for, respectively, the linear, inverse and inverse square models).

The relative spike frequency at amplitude $a$ and mean spike variability $v$ is given by

$$h\left(a,v\right)=\sum_{j} \frac{1}{2\pi\sigma_{a}\left(j\right)\sigma_{v}\left(j\right)}e^{-\frac{\left(a-\mu_{a}\left(j\right)\right)}{2\sigma_{a}\left(j\right)^{2}}}e^{-\frac{\left(v-\mu_{v}\left(j\right)\right)}{2\sigma_{v}\left(j\right)^{2}}}$$

where the total number of neurons depends on the brain region’s cell density (see main text for details). We obtained $\mu_{a}\left(j\right)$ from $r_{j}$ using each of the three fitted models in S3.

To simulate a recording where the cell of interest has a known SNR but unknown radial distance from the recording electrode, the normalised mean amplitude is given by

$$\widehat{\frac{\mu_{a}}{\sigma_{\varepsilon}}}=\frac{1}{\sqrt{\widehat{\nu}^{2}-\mu_{\gamma}^{2}}}=\sqrt{\frac{SNR^{2}-1}{k^{2}\left(1+\mu_{\gamma}^{2}\right)}}$$

For convenience, the amplitude is normalised by the noise $\sigma_{\varepsilon}$. Also,

$$\sigma_{a}\left(j\right)=\mu_{\gamma}\mu_{a}\left(j\right)$$

Assuming a spherically symmetric Gaussian distribution in variability space (*i.e.* accounting for noise-related shape variability only),

$$\sigma_{v}\left(j\right)=\sqrt{\frac{\pi}{2}}\left(1-\sqrt{1-\frac{1}{SNR_{j}^{2}}}\right)$$

where for the *j*^th^ neuron, $r_{j}$ is calculated by randomly scattering neurons in a 300 μm hemisphere (centred at the electrode tip) to yield an average density of $\rho$.

In simulations, $\mu_{v}\left(j\right)$ was randomly drawn (with replacement) from the matrix of pair-wise spike variability values from our dataset. This was done to avoid needing to explicitly model the distribution of average spike variability values. Only biphasic spikes were included. In an actual recording, neurons *within* a brain region may have more similar biological characteristics than occurs *between* cells in different brain regions, raising the possibility that spikes from different neurons within an actual recording have very similar intrinsic spike shapes. If so, the theoretical results here would underestimate the spike misclassification rate.

As not all cells in a brain region are active during any one recording, the number of possible cells whose spikes may be misclassified as belonging to the cell of interest will vary. Clearly, the fewer the number of active cells in a recordable volume, the lower the relative frequency of spikes which may appear similar to those of the cell of interest. The fraction of active cells was modelled as a scale factor, *i.e.*,

$$h^{*}\left(a,v\right)=k_{f}h\left(a,v\right)$$

This therefore represents the average relative frequency distribution in amplitude shape space over many experiments. Hence the spike misclassification rate can be considered as a pooled probability over many similar recordings, with associated variability from one experiment to the next.

For each SNR, the Z-score in amplitude-shape space which minimised the overall classification error was found (at 0.05 resolution). The reported $P_{mis}$ value corresponds to the optimal Z-score for each SNR (see Figures S5, S7 and S9 for, respectively, the linear, inverse and inverse square models), and therefore underestimates the true misclassification rate if a standard clustering Z-score value is used for all recordings. See Tables S4 and S6 for the minimum SNRs required to achieve given neuron classification success rates for, respectively, the inverse and inverse square models.

Simulation details: $h^{*}\left(a,v\right)$ was averaged over 100 trials, with maximum simulated radial distance of 300μm for inverse and inverse square models, and 143μm for linear model (X-intercept); $P_{mis}$ was calculated using $k_{f}\in\left\{1,0.1,0.01\right\}$ for each test $SNR_{a}\in\left\{1,2,\cdots,35\right\}$, for both high and low cell density regions. NB: due to the assumption that all true spikes are detected regardless of SNR, there is an artefact at SNRs very close to unity (noise). This occurs because of the practical need to limit the radial distance in simulation, whereas at SNR→1, the inverse-square model expects r→∞. If there is unlimited brain volume, this means an unbounded number of cells at SNR→1. In practice, extracellular spike detection at extremely low SNRs is unreliable and is typically excluded from further analysis.
